# One-step synthesis of ball-shaped metal complexes with a main absorption band in the near-IR region

**DOI:** 10.1038/s41598-019-53014-7

**Published:** 2019-11-11

**Authors:** Taniyuki Furuyama, Fumika Shimasaki, Natsumi Saikawa, Hajime Maeda, Masahito Segi

**Affiliations:** 1 Graduate School of Natural Science and Technology, Kanazawa University, Kakuma-machi, Kanazawa, 920-1192 Japan; 2 Japan Science and Technology Agency (JST)-PRESTO, 4-1-8 Honcho, Kawaguchi, Saitama, 332-0012 Japan

**Keywords:** Structure elucidation, Ligands

## Abstract

The design of near-IR materials is highly relevant to energy and pharmaceutical sciences due to the high proportion of near-IR irradiation in the solar spectrum and the high penetration of near-IR light in biological samples. Here, we show the one-step synthesis of hexacoordinated ruthenium and iron complexes that exhibit a main absorption band in the near-IR region. For that purpose, novel tridentate ligands were prepared by condensation of two diimines and four cyanoaryl derivatives in the presence of ruthenium and iron template ions. This method was applied to a wide variety of cyanoaryl, diimine, and metal ion combinations. The relationship between the structure and the optical and electrochemical properties in the resulting complexes was examined, and the results demonstrated that these compounds represent novel near-IR materials whose physical properties can be controlled based on rational design guidelines. The intense absorption bands in the 700–900 nm region were assigned to metal-to-ligand charge transfer (MLCT) transitions, which should allow applications in materials with triplet excited states under irradiation with near-IR light.

## Introduction

Light-harvesting materials play a prominent role in various fields of materials sciences, such as solar energy conversion^1,2^, artificial photosynthesis^3,4^, environmental analysis^5^, and therapeutics^6,7^. Organic materials present advantages over inorganic materials in terms of the diversity of the structures available, their flexibility, and the relatively low cost of their synthesis. Light is typically classified by its wavelength, and visible-light-harvesting materials usually exhibit bright colors, and have thus attracted great interest for a long time. On the other hand, near-IR light (especially the 700–1000 nm region) is “invisible” and its practical applications have remained undeveloped. However, the characteristics of near-IR light are attractive for applications in advanced functional materials. For instance, half the density of solar energy is composed of near-IR light. The high penetration of human tissue of light in this spectral region (the so-called therapeutic windows) moreover suggests relatively low toxicity and high selectivity for biological applications. Hence, a wide range of potential applications of near-IR materials has been proposed, such as in dye-sensitized solar cells (DSSCs)^8,9^, organic photovoltaics (OPVs)^10,11^, photocatalysts^12–14^, photosensitizing agents^15,16^, n-type semiconductors^17,18^, biological imaging^19–22^, and cancer treatment^23–25^.

**Figure 1:**
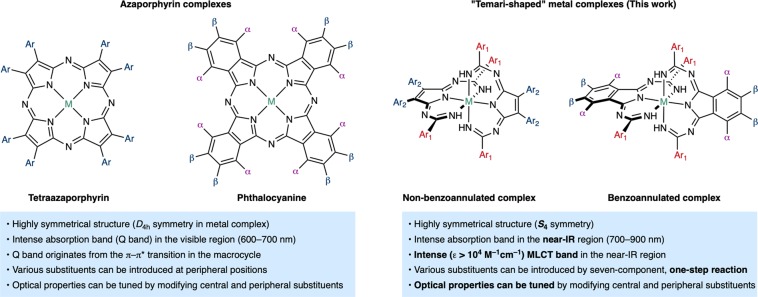
General properties of azaporphyrins and the metal complexes in this work.

Herein, we report the synthesis and characterization of a series of Temari (a traditional hand-made Japanese ball)-shaped metal complexes that absorb in the near-IR window (Fig. 1). For the development of near-IR materials, a synthetically facile organic “platform” with robust, finely tunable, and predictable properties would be highly desirable; however, so far, this puzzle piece remains missing in organic materials chemistry. Phthalocyanine (Pc)-metal complexes present highly symmetrical *D*_4h_ structures with an intense absorption band in the visible region (the so-called Q band), whose properties can be tuned by modifications on the Pc skeleton^26^. Heavy-atom effects of the metal center are also important for optical materials that exhibit triplet excited states. Currently, Pc derivatives are employed in optical and electronic materials under visible light in various fields^27,28^. On the other hand, the typical Pc Q band can be assigned to π–π* transitions in the Pc macrocycle, indicating that orbital effects of the central heavy metal are relatively small. Ruthenium polypyridine complexes are well-known as efficient optical materials^29^. Metal-to-ligand charge transfer (MLCT) transitions in the lower energy region are often essential for the unique optical properties of such Ru(II) complexes. Although a number of Ru(II) complexes have been designed to reduce the energy of MLCT transitions, which is commensurate with a bathochromic shift of the absorption^8,30–32^, a general strategy toward compounds able to exploit the near-IR light (>700 nm) with high efficiency (*ε* > 10^4^ M^−1^ cm^−1^) remains elusive. Various ligands for Ru(II) complexes have been synthesized, most of them by literature procedures. We believe that structurally novel organic ligands synthesized by newly developed methods will provide the opportunity to overcome the aforementioned unresolved problems. Recently, several automated processes for the development of new organic synthetic methods have been proposed, albeit that they require expert chemistry knowledge and specialized training^33,34^. Regardless, the design of unusual reactions and compounds by synthetic chemistry experts remains an important topic in synthetic organic chemistry.

## Results

### Synthesis of hexacoordinated complexes of Fe and Ru

**Figure 2:**
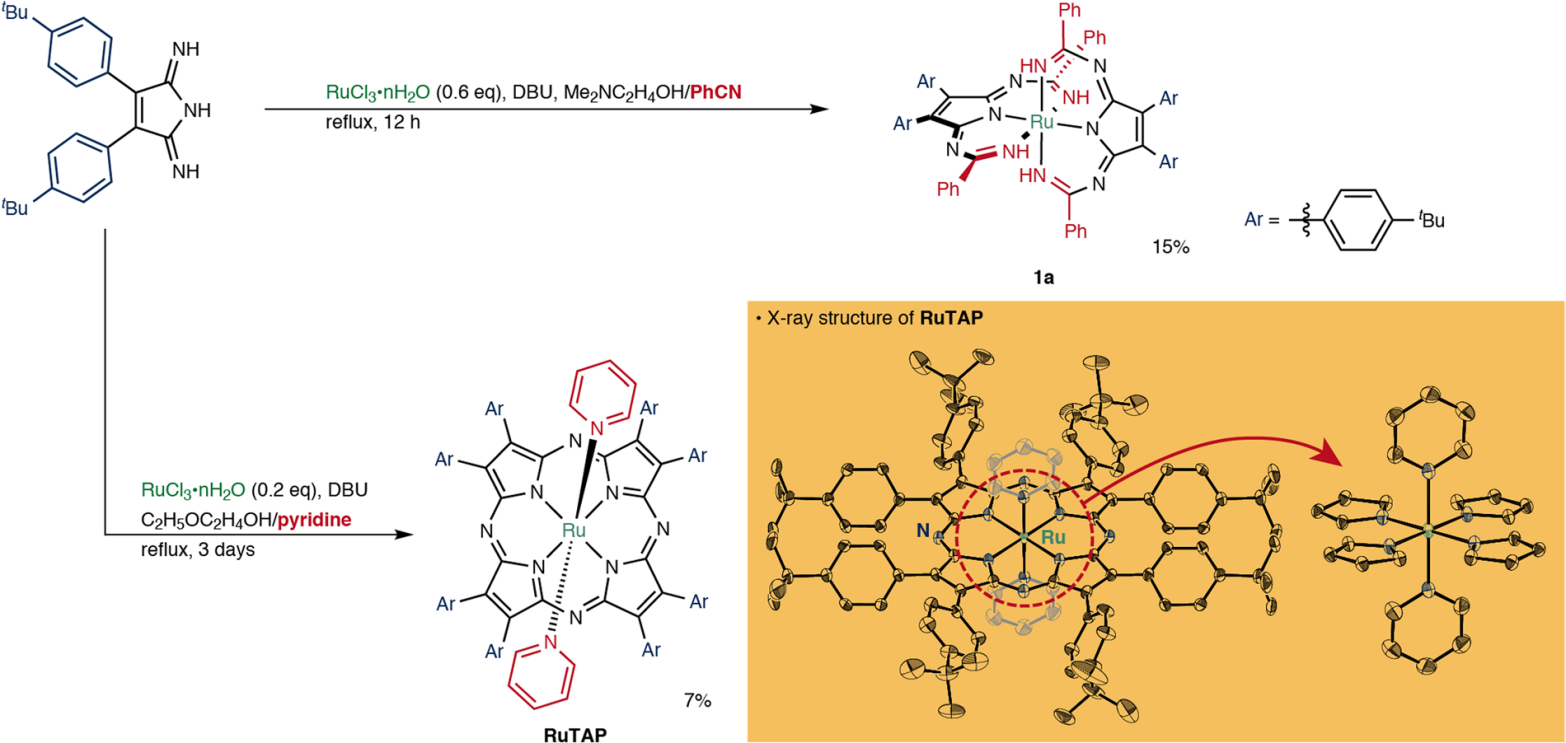
Synthesis of **RuTAP** and **1a**.

**Figure 3:**
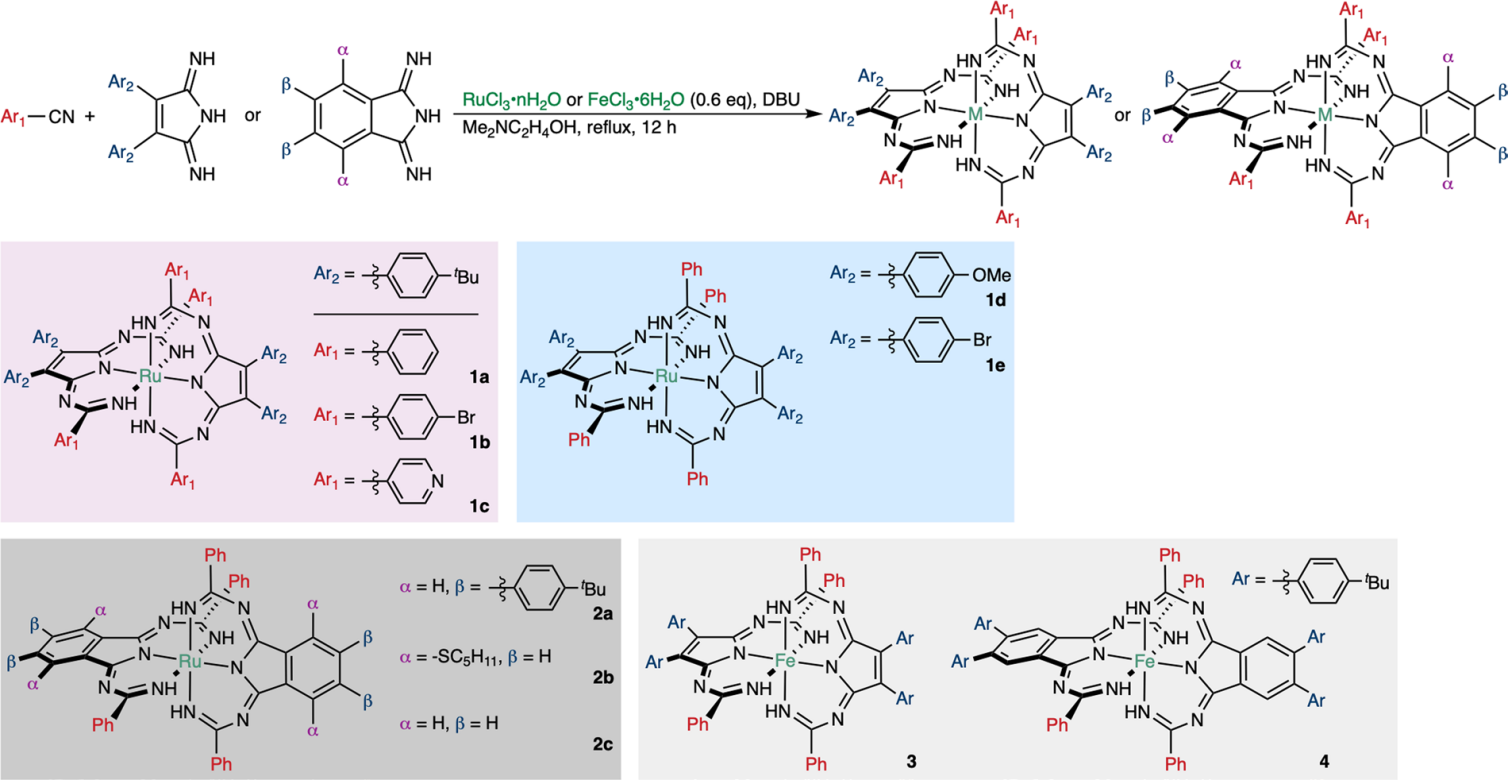
General scope of the seven-component condensation reaction between cyanoaryls, diimines, and metal salts.

Pc ruthenium complexes (RuPc) bearing one or two axial ligands were synthesized by a direct condensation or by ligand-substitution reactions using suitable Pc precursors^35^. We have previously reported the unique optical properties of octaaryl-substituted tetraazaporphyrin (TAP) phosphorus(V) complexes^36,37^, and thus TAP ruthenium complexes (RuTAPs) were chosen as our initial synthetic targets. A RuTAP complex bearing an axial pyridyl ligand (**RuTAP**) was synthesized from the corresponding pyrroline-diimine derivatives, ruthenium trichloride, and pyridine, following a modified literature procedure^38^. On the other hand, the synthesis of RuTAP complexes bearing an axial cyanophenyl ligand was unsuccessful when pyrroline-diimine and RuCl_3_ were condensed in the presence of benzonitrile instead of pyridine^39^. Interestingly, an unexpected moss-green compound (**1a**) was isolated instead of RuTAP. A detailed structural characterization (*vide infra*) revealed that **1a** exhibits a novel ball-shaped structure composed of one octahedral hexacoordinated ruthenium and two tridentate ligands (Fig. 2). The tridentate ligand consists of one diimine and two benzonitrile moieties; therefore, **1a** was generated in a seven-component one-step reaction. The robustness of this structure was confirmed by changing the ligands and central metal. Figure 3 illustrates the generality of the synthesis, and the optimization of the reaction conditions is summarized in Supplementary Table 4. Both cyanoaryl (Ar_1_ position) and diimine (Ar_2_ position) compounds were successfully transformed. When 4-cyanopyridine was used, only **1c** was obtained, while 4-cyanopyridine-coordinated RuTAP was not observed. Moreover, benzoannulated complexes **2a–c** were obtained from the corresponding (un)substituted diiminoisoindoline derivatives. This synthetic method can also be applied to the synthesis of iron complexes. Both non-benzoannulated (**3**) and benzoannulated (**4**) complexes were obtained using FeCl_3_ as the metal template.

**Figure 4:**
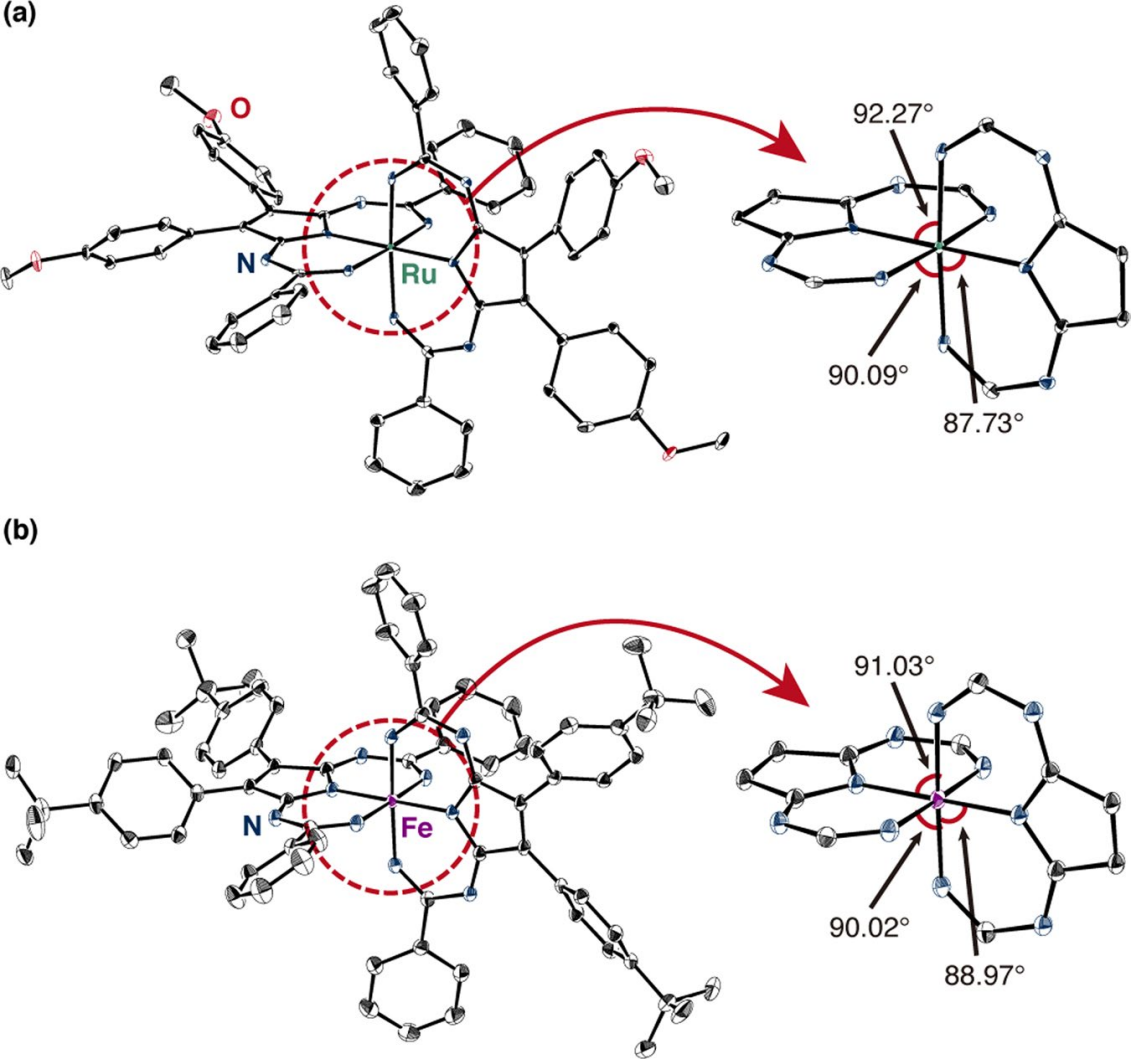
X-ray crystal structure of (**a**) ruthenium complex **1d** and (**b**) iron complex **3**. Thermal ellipsoids are shown at 50% probability, and hydrogen atoms as well as solvent molecules have been omitted for clarity.

These compounds display excellent solubility in common organic solvents, and were fully characterized by NMR spectroscopy, HR-MALDI-TF-ICR mass spectrometry and elemental analysis. For instance, the ^1^H NMR signals derived from the cyanoaryl (Ar_1_) and peripheral aryl groups (Ar_2_) of the diimine units of **1a** appear as sharp peaks in the aromatic region (7–8 ppm), supporting the notion that these compounds exhibit a four-fold symmetric structure and diamagnetic character (Supplementary Fig. 1). A downfield singlet ( > 9 ppm) could be assigned to the imine proton of the cyanoaryl moiety. The structures of Temari-shaped complexes **1d** (Ru) and **3** (Fe) were unambiguously determined by single-crystal X-ray diffraction analysis (Fig. 4). The symmetry of their unit cells is tetragonal with a highly symmetrical *S*_4_ structure. No residual peaks remained after the signals for the complex and the solvent were assigned, indicating that these complexes are neutral. Considering the results of the NMR and X-ray diffraction measurements suggests that the octahedral-hexacoordinated central metals are in the oxidation state + II. The chelation angles weakly deviate from perfect octahedral coordination due to the unsymmetric nature of the ligand. The ligands in the Ru and Fe complexes present similar bond lengths (Supplementary Fig. 2). Although the structure of the auxiliary ligand is rigid and planar, significant bond length alternation was observed. The bond length between the central metal and nitrogen is consistent with typical M–X bonds of hexacoordinated metal complexes^40,41^. Therefore, other metals that favor octahedral hexacoordinated structures might also be used for the synthesis of corresponding Temari-shaped complexes.

### Near-IR optical and electrochemical properties

**Figure 5:**
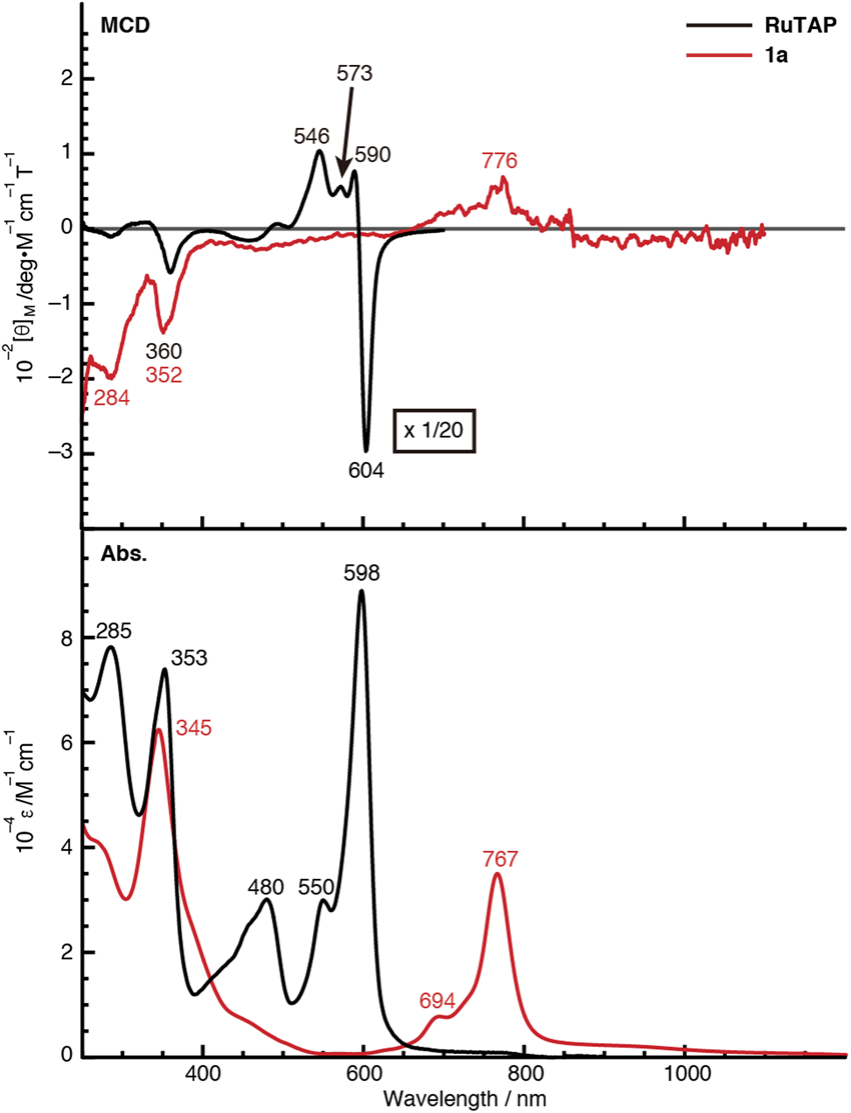
UV-vis-NIR MCD (top) and absorption (bottom) spectra of **RuTAP** and **1a** in CHCl_3_.

The absorption and magnetic circular dichroism (MCD) spectra of **RuTAP** and **1a** are shown in Fig. 5. The Q bands of typical Pc and TAP ruthenium complexes appear in the range of 600–700 nm^35^. Interestingly, the sharp (full width at half maximum (fwhm) = 680 cm^−1^) and intense (*ε* = 3.5 × 10^4^ M^−1^ cm^−1^) absorption band of **1a** appears in the near-IR region beyond 700 nm (e.g. 767 nm in chloroform), despite its non-cyclic structure. The MCD intensity indicates changes in the orbital angular momentum between the ground and excited states^42^. For instance, the intense dispersion-type Faraday *A* terms (604 and 590 nm) in the Q band region of **RuTAP** indicate a large angular momentum change (±9) derived from π–π* transitions in the TAP macrocycle. On the other hand, a very weak MCD signal (776 nm) was found for **1a** in the near-IR region, indicating a small change in angular momentum. Hence, it may be concluded that the intense absorption peak does not correspond to a simple π–π* transition in the ligand. Small solvent effects were also observed in the absorption spectra of **1a** in various solvents (Supplementary Fig. 3). A detailed assignment is provided below. Despite the intense absorption in the near-IR region, the photostability of the complexes is very high. A CHCl_3_ solution of **1a** showed no photobleaching ( < 5%) after irradiation for 22 h (Supplementary Fig. 4).

**Figure 6:**
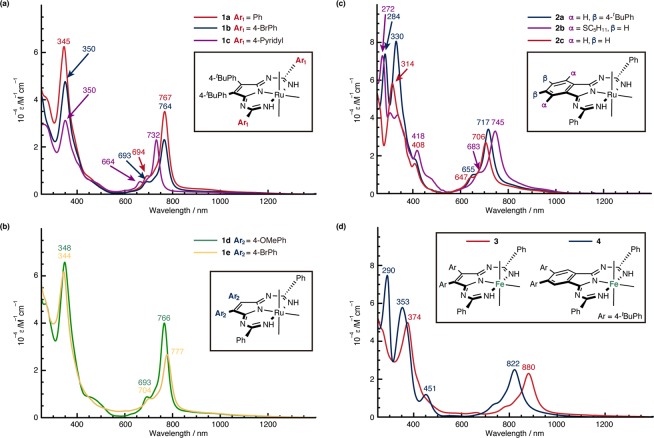
UV-vis-NIR absorption spectra in CHCl_3_ of Ru complexes upon (**a**) changes at the Ar_1_ position, (**b**) changes at the Ar_2_ position, (**c**) benzoannulation, and (**d**) of iron complexes.

Figure 6 shows the absorption spectra of the Temari-shaped complexes synthesized in this work. Structural effects were rationalized in terms of the three types of components: (1) Peripheral substituent effects (Ar_1_ and Ar_2_ moieties), (2) benzoannulation effects, and (3) central metal effects (Ru vs Fe). The intense near-IR band was maintained (760–780 nm) upon changing the peripheral substituents to both electron-donating (**1d**) and electron-withdrawing (**1b**, **1e**) groups, while electron-deficient pyridyl-substituted **1c** showed a blue-shifted band (732 nm). This trend resembles the peripheral substituent effects of typical TAP metal complexes^43,44^, since these groups do not participate in the π-conjugation of the ligands. Although the Q band of typical azaporphyrin derivatives is red-shifted upon benzoannulation^45^, the near-IR band of the Temari-type complexes is blue-shifted upon benzoannulation (Fig. 6c). The introduction of electron-donating groups at suitable positions of Pc is an efficient approach to red-shift the Q band (by ca. 100 nm)^46,47^, while substituent effects on the benzoannulated complexes are relatively small (**2a** vs **2c**: 11 nm, 220 cm^−1^, **2b** vs **2c**: 39 nm, 740 cm^−1^). The effects of changing the central metal are more significant than those arising from changing the peripheral substituents or from benzoannulation (Fig. 6d). The Fe complexes with both non-benzoannulated (**3**) and benzoannulated (**4**) units exhibit a red-shift of ca. 110 nm (ca. 1700 cm^−1^) compared to the bands of the corresponding Ru complexes (**1a** and **2a**). Moreover, the shoulder of the absorption band of **3** reached 900 nm. This central-metal effect clearly indicates that the orbitals of the metal contribute to these intense near-IR bands.

**Figure 7:**
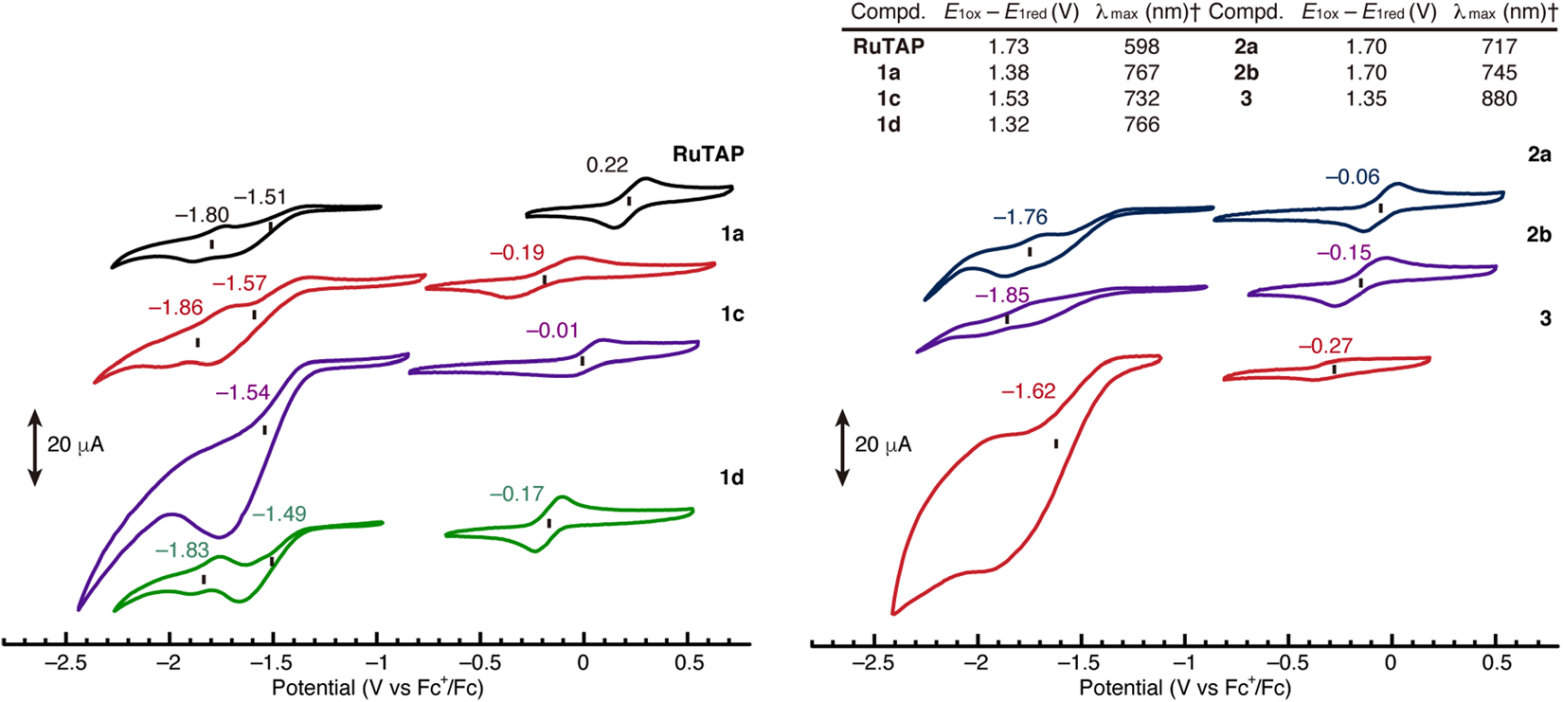
Cyclic voltammograms of **RuTAP**, **1a**, **1c**, **1d, 2a**, **2b**, and **3**; [analyte] = 0.5 mM; solvent: THF; supporting electrolyte: 0.1 M [^*n*^Bu_4_N][ClO_4_]. All potentials are referenced to the ferrocene/ferrocenium couple. ^†^In CHCl_3_.

The cyclic voltammograms (CVs) of **RuTAP**, **1a**, **1c**, **1d**, **2a**, **2b**, and **3** were measured in THF, and representative curves are shown in Fig. 7. It is well established for azaporphyrins that the potential difference between the first oxidation and reduction potentials (*E*_1ox_ − *E*_1red_) is correlated to the lowest transition energy^48^. All complexes present one or two reduction and oxidation processes. The reduction waves of some complexes are partly irreversible, so that their redox potentials were assigned by using corresponding differential pulse voltammograms (DPVs) (Supplementary Fig. 5). The value of *E*_1ox_ − *E*_1red_ decreases in the order **RuTAP** > **2a** ~ **2b** > **1c** > **1a** > **3**, supporting the position of the intense absorption band in the visible-to-near-IR region. An anodic shift (0.18 V) of the oxidation potential was observed upon changing the Ar_1_ substituent (from **1a** to **1c**), while changing the Ar_2_ group (from **1a** to **1d**) only marginally influenced the oxidation potential. Upon benzoannulation (from **1a** to **2a**), the first *reduction* couple of **2a** exhibits a cathodic shift of 0.19 V compared to the first reduction couple (−1.57 V) of **1a**, i.e., the LUMO is destabilized by the benzoannulation. On the other hand, the first *oxidation* couple of **3** shows a cathodic shift of 0.08 V compared to the first oxidation couple (−0.19 V) of **1a**. Hence, the red-shift of the near-IR band of Fe complexes can be assigned to a destabilization of the HOMO.

### Rationalizing the structure–property relationships

**Figure 8:**
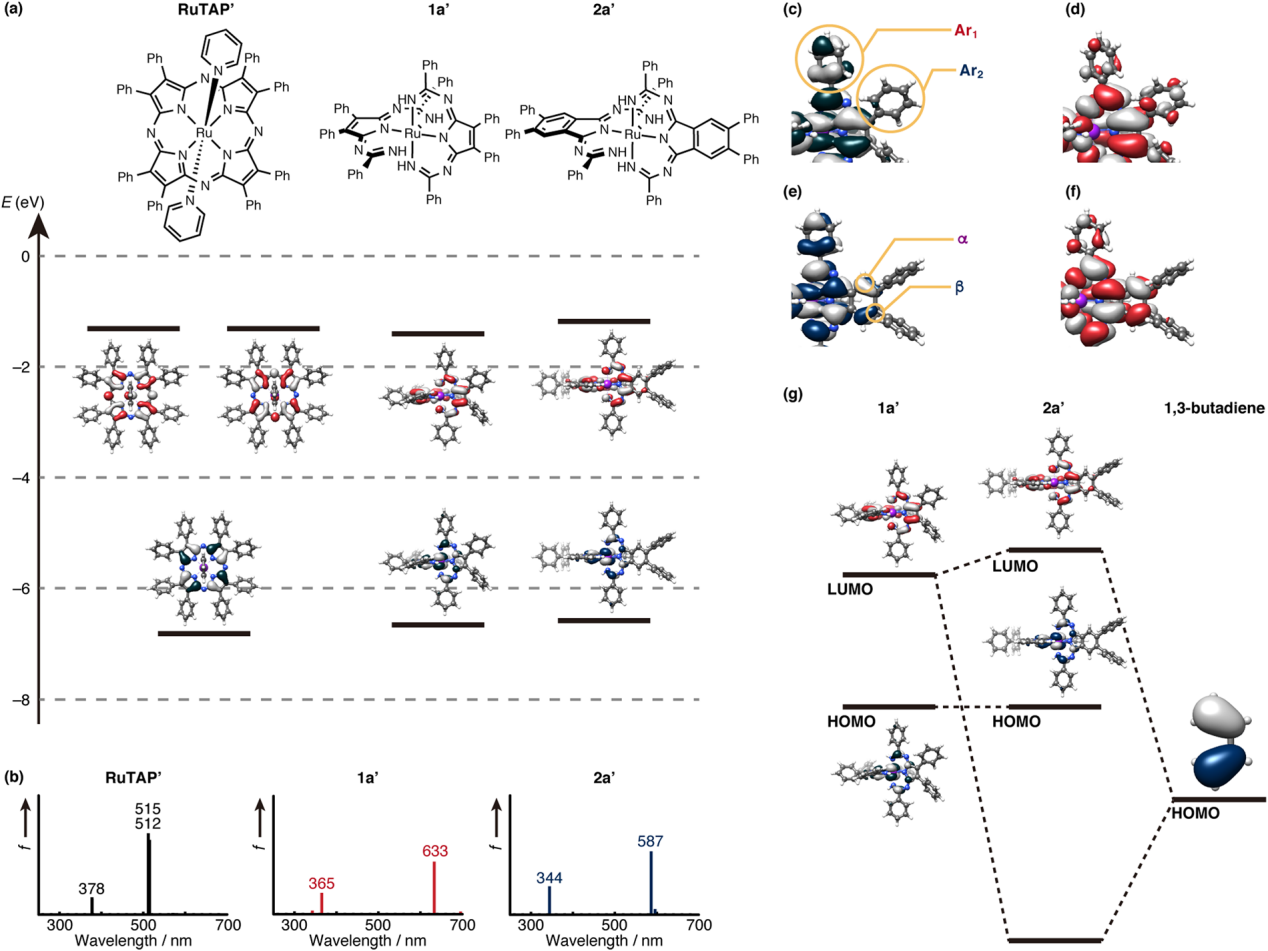
(**a**) Partial molecular energy diagram and orbitals of **RuTAP’**, **1a’**, and **2a’** as well as (**b**) their calculated absorption spectra. Blue and red plots indicate occupied and unoccupied MOs, respectively. (**c**) Magnified HOMO of **1a’**. (**d**) Magnified LUMO of **1a’**. (**e**) Magnified HOMO of **2a’**. (**f**) Magnified LUMO of **2a’**. (**g**) Qualitative MO diagrams of the frontier orbitals of **1a’** and **2a’** and the HOMO of 1,3-butadiene. The MO isovalues are 0.030 in panels a and g, and 0.010 in panels c–f. Calculations were carried out at the ωB97XD/631SDD//B3LYP/631SDD level of theory, using the polarizable continuum model (PCM) that mimicked the solvation effect of CHCl_3_ (for details, see the Supplementary Information).

Based on the experimental optical and electrochemical results, we attempted to rationalize the structure–property relationships in these Temari-shaped complexes by performing molecular-orbital (MO) calculations. Model complexes **RuTAP’** (tetraazaporphyrin), **1a’** (non-benzoannulated), and **2a’** (benzoannulated) were used, where the peripheral *p*-*tert*-butylphenyl substituents were replaced by phenyl groups in the interest of simplicity. The optimized structures were consistent with those determined from the experimental spectroscopy measurements (Supplementary Fig. 6). The partial MO energy diagrams of the model structures and calculated absorption spectra are shown in Fig. 8a,b, and the results of time-dependent density functional theory (TD-DFT) calculations are summarized in Supplementary Table 5. The calculated intense absorption bands in the longer wavelength regions for all compounds correspond to HOMO–LUMO transitions. The frontier orbitals of **RuTAP’** follow the Gouterman “four-orbital” model^49^, i.e., these orbitals correspond to a_1u_-, e_gy_-, and e_gx_ orbitals. Therefore, the calculated transition at 508 nm can be assigned to the experimental Q band as a π–π* transition, while the contribution of the central ruthenium atom is small.

The calculated transition of **1a’** at 633 nm supports the intense absorption band of **1a** appearing at a longer wavelength than that of **RuTAP** (the calculated absorption bands for **RuTAP’** are 515 and 512 nm). The HOMO of **1a’** is located on the d-orbital of the central ruthenium atom, while the LUMO is localized on the ligand. Hence, the HOMO–LUMO transition can be assigned to an MLCT transition. The MCD and CV results support this conclusion. The weak MCD signal indicates that the transition has CT character with small changes in the angular momentum^37^. Since the oxidation potential contributes to the HOMO energy level, the oxidation potential of Fe complex **3** shifts cathodically with a smaller *E*_1ox_ − *E*_1red_ gap.

Although the peripheral substituent effects were relatively small, these effects were clarified by careful inspection of the MOs of model complexes. In terms of the electronic contributions to the HOMO of **1a’**, a weak contribution from the Ar_1_ position was observed (Fig. 8c). In the case of **1c**, the electron-deficient pyridyl groups stabilize the HOMO of the complex. Therefore, the band gap of **1c** increases and a blue-shifted absorption band is observed. The first oxidation couple in the voltammogram of **1c** (−0.01 V) appears more anodically shifted than that of **1a**, confirming that the heterocycles influence the HOMO of the complexes. The calculated absorption spectrum of the model complex **1c’** also supported the differences (Supplementary Fig. 7). On the other hand, the contribution from the Ar_2_ positions is small for both the HOMO and LUMO (Fig. 8d), indicating that the physical properties depend only marginally on the diimine substituents. For the benzoannulated complexes, the contribution from the annulated benzene moieties to the LUMO is larger than that to the HOMO (Fig. 8e,f). In the case of Pc, the introduction of electron-donating groups destabilizes the HOMO^46^, while electron-donating substituents stabilize the LUMO, in agreement with the experimental absorption spectra of **2a** and **2b**.

The blue-shift of the intense near-IR band upon benzoannulation was also reproduced by the calculations. The LUMO of **2a’** was destabilized, supporting a cathodic shift of the reduction potential of **2a**. The changes in MO energy can be explained by an MO diagram based on a simple theory^50,51^. Benzoannulation can be interpreted as an MO interaction between parent **1a’** and 1,3-butadiene (Fig. 8g). The symmetry of the interacting orbitals determines whether the orbitals are able to interact or not. The HOMO of 1,3-butadiene favors cooperative interactions with the appropriate position of the LUMO of **1a’**, leading to an occupied bonding MO (not shown) and an unoccupied antibonding orbital (LUMO of **2a’**), while the HOMO of **1a’** cannot interact with the MO of 1,3-butadiene due to a mismatch of symmetry. The net effect of these interactions is an unaltered HOMO and a destabilized LUMO, resulting in a blue-shifted MLCT absorption upon benzoannulation of **1a’**.

## Discussion

**Figure 9:**
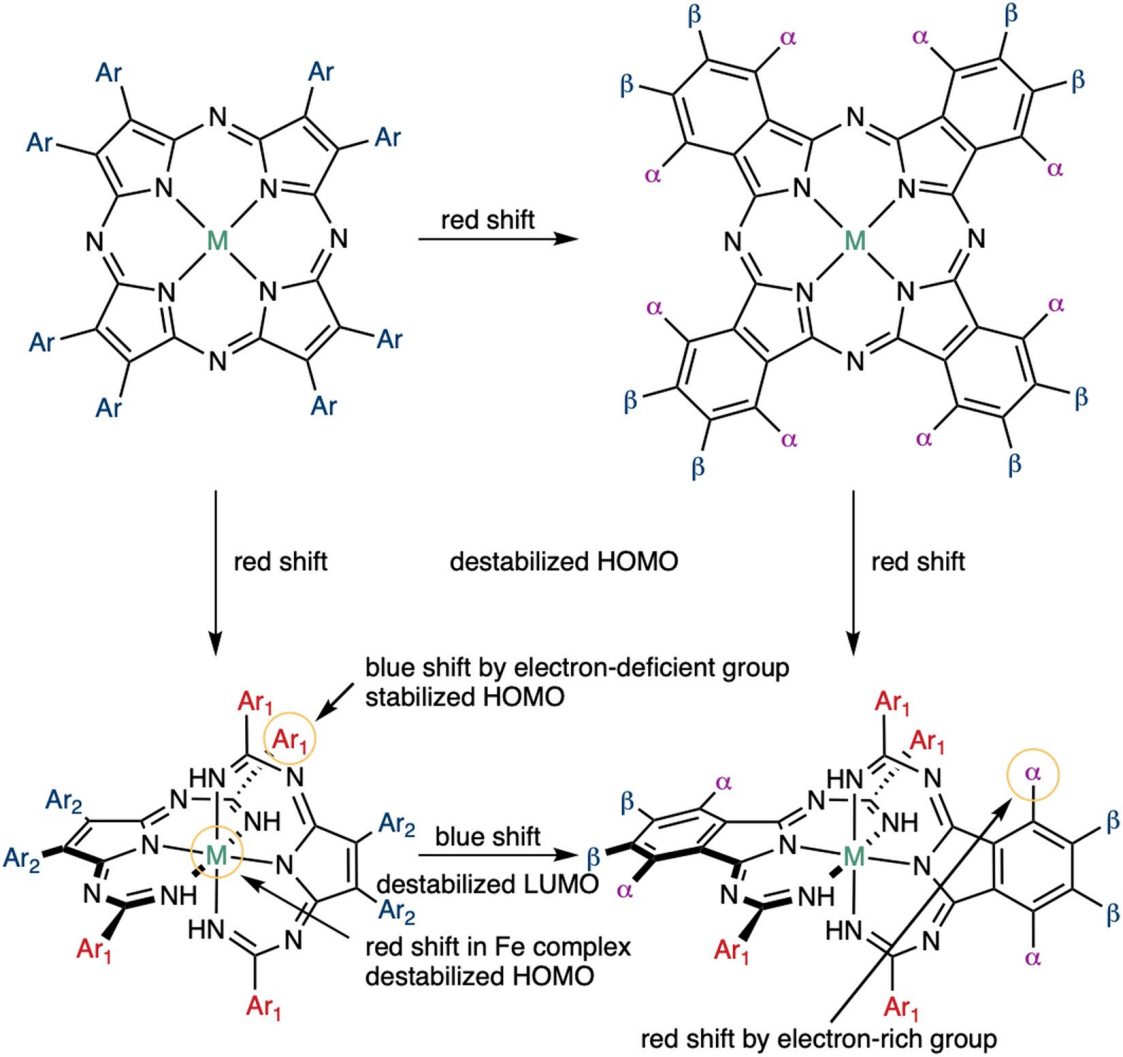
Summary of the substituent effects on the physical properties.

The one-step condensation of cyanoaryls and diimines in the presence of a metal (Ru or Fe) afforded novel ball-shaped (Temari) complexes that absorb near-IR light beyond 700 nm. Various azaporphyrin precursors could be used in this synthesis and a series of compounds with various substituent groups/benzoannulated moieties/central metals were successfully prepared and characterized. MCD spectra and theoretical calculations revealed that MLCT transitions significantly contribute to this near-IR absorption band. As summarized in Fig. 9, the effects of the peripheral substituents and central metal on the optical and electrochemical properties were rationalized based on a combination of theoretical calculations and experimental results. These features highlight the advantages of azaporphyrins (Fig. 1) for industrial applications, which are currently under investigation. However, the concept underlying the synthetic strategy in this work should open the door to a variety of applications of metal-based near-IR materials.

## Methods

### Typical procedure for the synthesis of Temari-shaped ruthenium complexes such as 1a

Under an Ar atmosphere, diimine **5a** (89.3 mg, 0.25 mmol) and ruthenium trichloride (31.5 mg, 0.15 mmol) were dissolved in benzonitrile (1.0 mL, 9.7 mmol) and dimethylaminoethanol (0.5 mL), followed by the addition of DBU (0.1 mL, 0.67 mmol). The mixture was stirred under reflux for 12 h, before it was concentrated. The residue was dissolved and extracted with CHCl_3_. The organic layer was washed with brine and dried over Na_2_SO_4_, filtered and concentrated *in vacuo*. The product was purified by column chromatography on silica gel (eluent: CHCl_3_/Hexane = 1/1 v/v) to provide **1a** as a moss-green solid (23.6 mg, 18.8 μmol, 15%) after recrystallization from methanol. ^1^H NMR (500 MHz, CDCl_3_, 1 mM): *δ* 9.53 (s, 4H, imine-N*H*), 7.98 (d, 8H, *J* = 8.5 Hz, pyrrole-^*t*^BuPh-Ph*H*), 7.80–7.78 (m, 8H, ArCN-Ph*H*), 7.50 (d, 8H, *J* = 8.5 Hz, pyrrole-^*t*^BuPh-Ph*H*), 7.29–7.26 (m, 12H, ArCN-Ph*H*), 1.41 (s, 36H, pyrrole-^*t*^BuPh-^*t*^Bu*H*); UV-Vis-NIR (CHCl_3_): *λ*_max_ = 767 nm (*ε* = 35000 M^−1^ cm^−1^) and 345 nm (*ε* = 63000 M^−1^ cm^−1^); HR-MS (MALDI-MS): *m*/*z* = 1230.5290, calcd. for (C_76_H_76_N_10_Ru)^+^ = 1230.5313 [(M)^+^].

### Theoretical calculations

DFT geometry optimizations were carried out using the B3LYP functional of the Gaussian 09 software package^52^. The Ru atoms were described using an SDD^53^. The 6–31G* basis set was used for all the other atoms (denoted as 631SDD). After geometry optimization, TD-DFT calculations were performed using ωB97XD^54^ and the same basis set. All calculations used a relatively simple self-consistent reaction field (SCRF) method based on the polarizable continuum model (PCM)^55^ that mimicked the solvation effect of chloroform (ε = 4.7113). For further details, calculated coordinates, and TD-DFT output, see the Supplementary Information.

## Supplementary information

Supplementary Infromation

## Data Availability

Crystallographic data (CIF files) for **RuTAP**, **1d**, and **3** have been deposited with the Cambridge Crystallographic Data Centre (CCDC) as supplementary publications under reference numbers CCDC 1892288 (**RuTAP**), CCDC 1892286 (**1d**), and CCDC 1892287 (**3**). These data can be obtained free of charge from the CCDC via www.ccdc.cam.ac.uk/data_request/cif. All other data supporting the findings of this study are available within the article and its Supplementary Information.
