# Supplementary material for: One-step synthesis of ball-shaped metal complexes with a main absorption band in the near-IR region
Source: Sci Rep. 2019 Nov 11;9:16528. doi: 10.1038/s41598-019-53014-7 (PMC6848132; doi:10.1038/s41598-019-53014-7)
Supplement: Supplementary file 1 — Supplementary Infromation [file 41598_2019_53014_MOESM1_ESM.pdf]

## Supplementary Information

# One-step Synthesis of Ball-shaped Metal Complexes with a Main Absorption Band in the Near-IR Region

Taniyuki Furuyama[*,1,2], Fumika Shimasaki[1], Natsumi Saikawa[1], Hajime Maeda[1], and Masahito Segi[1]

[1]Graduate School of Natural Science and Technology, Kanazawa University, Kakuma-machi, Kanazawa, 920-1192, Japan
[2]Japan Science and Technology Agency (JST)-PRESTO, 4-1-8 Honcho, Kawaguchi, Saitama, 332-0012, Japan

*E-mail: tfuruyama@se.kanazawa-u.ac.jp

## Table of Contents

General Comments S2

Additional Experimental and Computational Results S7

Full Experimental Procedures S15

Copies of the NMR Spectra of Studied Compounds S27

Full Computational Details S38

References S51

## General Comments

NMR spectra were obtained on a JEOL ECA-500 spectrometer. Chemical shifts are expressed in δ (ppm) values, and coupling constants are expressed in hertz (Hz). $^1$H-NMR spectra were referenced to the residual solvent as an internal standard. The following abbreviations are used: s = singlet, d = doublet, m = multiplet, and brs = broad singlet. High-resolution mass spectra (HRMS) were recorded on a Bruker Daltonics solariX spectrometer (MALDI). Electronic absorption spectra were recorded on a JASCO V-570 spectrophotometer. Magnetic circular dichroism (MCD) spectra were obtained on a JASCO J-725 spectrodichrometer equipped with a JASCO electromagnet capable of producing magnetic fields of up to 1.03 T (1 T = 1 tesla) with both parallel and antiparallel fields. The magnitudes were expressed in terms of molar ellipticity per tesla ($[\theta]_M$ / deg $dm^3 mol^{-1} cm^{-1} T^{-1}$). CV measurements were recorded with a Hokuto Denko HZ5000 potentiostat under nitrogen atmosphere in THF solutions with 0.1 M of tetrabutylammonium perchlorate (TBAP) as a supporting electrolyte. Measurements were made with a glassy carbon electrode (area = 0.07 $cm^2$), an Ag/AgCl reference electrode, and a Pt wire counter electrode. The concentration of the solution was fixed at 0.5 mM and the sweep rates were set to 100 mV/s. The ferrocenium/ferrocene ($Fc^+$/Fc) couple was used as an internal standard.

## Crystallographic data collection

Data collection for **RuTAP**, **1d**, and **3** were carried out on a Bruker APEXIII CCD diffractometer with Bruker Helios multilayered confocal mirror monochromatized Cu*K*α radiation (λ = 1.54178 Å) at –183°C. The structures were solved by a direct method (SIR2004) [i] and refined using a full-matrix least square technique (SHELXL-2014). [ii] Yadokari-XG 2009 software was used as a GUI for SHELXL-2014.[iii] All non-hydrogen atoms were refined anisotropically. Positions of all hydrogen atoms were calculated geometrically, and refined by applying riding models. A part of peripheral substituents and solvents were severely disordered despite the low measurement temperature. Therefore, the structure was refined under thermally and positionally restrained conditions, using DFIX, SIMU, ISOR, and DELU commands. CCDC-1892288, 1892286 and 1892287 contains the supplementary crystallographic data for **RuTAP**, **1d**, and **3**, respectively. Their data can be obtained free of charge from Crystallographic Data Centre via www.ccdc.cam.ac.uk/data_request/cif.

**Supplementary Table 1.** Crystal data and structure refinement for **RuTAP**.

| | | |
|---|---|---|
| Empirical formula | $C_{134}H_{146}N_{10}Ru$ | |
| Formula weight | 1997.67 | |
| Temperature | 90(2) K | |
| Wavelength | 1.54178 Å | |
| Crystal system | Triclinic | |
| Space group | *P*–1 (No. 2) | |
| Unit cell dimensions | $a$ = 12.2234(5) Å | $\alpha$ = 78.8600(10)° |
| | $b$ = 14.6135(6) Å | $\beta$ = 79.064(2)° |
| | $c$ = 16.4982(6) Å | $\gamma$ = 73.0710(10)° |
| Volume | 2738.09(19) Å$^3$ | |
| *Z* | 1 | |
| Density (Calcd.) | 1.212 Mg/m$^3$ | |
| Absorption coefficient | 1.584 mm$^{-1}$ | |
| *F*(000) | 1064 | |
| Crystal size | 0.10 x 0.10 x 0.10 mm$^3$ | |
| Theta range for data collection | 7.409 to 68.586° | |
| Index ranges | -14<=h<=14, -17<=k<=17, -19<=l<=19 | |
| Reflections collected | 35414 | |
| Independent reflections | 9786 [*R*(int) = 0.0212] | |
| Completeness to theta = 67.679° | 97.3% | |
| Refinement method | Full-matrix least-squares on $F^2$ | |
| Data / restraints /parameters | 9786 / 304 / 901 | |
| Goodness-of-fit on $F^2$ | 1.061 | |
| Final *R* indices [$I > 2\text{sigma}(I)$] | $R_1$ = 0.0404, $wR_2$ = 0.1030 | |
| *R* indices (all data) | $R_1$ = 0.0417, $wR_2$ = 0.1048 | |
| Largest diff. peak and hole | 0.614 and -0.581 e.Å$^{-3}$ | |
| CCDC No. | 1892288 | |

**Supplementary Table 2.** Crystal data and structure refinement for **1d**.

| | |
|---|---|
| Empirical formula | $C_{35}H_{29}Cl_9N_5O_2Ru_{0.50}$ |
| Formula weight | 921.22 |
| Temperature | 90(2) K |
| Wavelength | 1.54178 Å |
| Crystal system | Tetragonal |
| Space group | *I*–4 (No. 82) |
| Unit cell dimensions | *a* = 21.8765(6) Å |
| | *b* = 21.8765(6) Å |
| | *c* = 15.9382(4) Å |
| Volume | 7627.7(5) $Å^3$ |
| *Z* | 8 |
| Density (Calcd.) | 1.604 $Mg/m^3$ |
| Absorption coefficient | 7.924 $mm^{-1}$ |
| *F*(000) | 3720 |
| Crystal size | 0.30 x 0.30 x 0.10 $mm^3$ |
| Theta range for data collection | 2.856 to 66.495° |
| Index ranges | -26<=h<=19, -25<=k<=20, -18<=l<=18 |
| Reflections collected | 16530 |
| Independent reflections | 6051 [*R*(int) = 0.0270] |
| Completeness to theta = 66.495° | 97.6% |
| Refinement method | Full-matrix least-squares on $F^2$ |
| Data / restraints /parameters | 6051 / 274 / 467 |
| Goodness-of-fit on $F^2$ | 1.038 |
| Final *R* indices [$I > 2\text{sigma}(I)$] | $R_1 = 0.0356$, $wR_2 = 0.0953$ |
| *R* indices (all data) | $R_1 = 0.0361$, $wR_2 = 0.0958$ |
| Largest diff. peak and hole | 1.155 and -0.647 e.$Å^{-3}$ |
| CCDC No. | 1892286 |

**Supplementary Table 3.** Crystal data and structure refinement for **3**.

| | |
|---|---|
| Empirical formula | $C_{78}H_{78}Cl_6FeN_{10}$ |
| Formula weight | 1424.05 |
| Temperature | 90(2) K |
| Wavelength | 1.54178 Å |
| Crystal system | Tetragonal |
| Space group | $I4_1/a$ (No. 88) |
| Unit cell dimensions | $a$ = 21.1202(5) Å |
| | $b$ = 21.1202(5) Å |
| | $c$ = 15.8410(4) Å |
| Volume | 7066.1(4) Å$^3$ |
| $Z$ | 4 |
| Density (Calcd.) | 1.339 Mg/m$^3$ |
| Absorption coefficient | 4.202 mm$^{-1}$ |
| $F$(000) | 2976 |
| Crystal size | 0.40 x 0.10 x 0.10 mm$^3$ |
| Theta range for data collection | 3.488 to 66.450° |
| Index ranges | -24<=h<=25, -25<=k<=25, -18<=l<=17 |
| Reflections collected | 25443 |
| Independent reflections | 3117 [$R$(int) = 0.0313] |
| Completeness to theta = 66.450° | 100.0% |
| Refinement method | Full-matrix least-squares on $F^2$ |
| Data / restraints /parameters | 3117 / 156 / 307 |
| Goodness-of-fit on $F^2$ | 1.080 |
| Final $R$ indices [$I > 2$sigma($I$)] | $R_1$ = 0.0522, $wR_2$ = 0.1496 |
| $R$ indices (all data) | $R_1$ = 0.0536, $wR_2$ = 0.1514 |
| Largest diff. peak and hole | 0.373 and -0.848 e.Å$^{-3}$ |
| CCDC No. | 1892287 |

## Additional Experimental and Computational Results

**Supplementary Table 4.** Reaction conditions of **1a** synthesis.

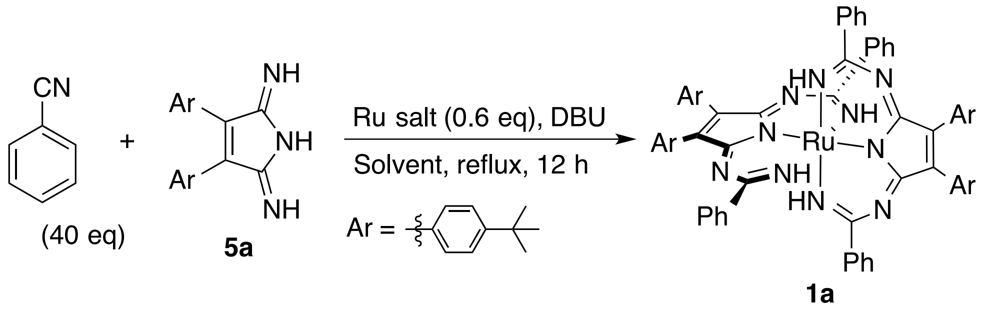


| Entry | Ru salt | Solvent | Yield (%)[a] |
|---|---|---|---|
| 1 | $RuCl_3$•n $H_2O$ | 2-Ethoxyethanol | 5 |
| 2 | $RuCl_3$•n $H_2O$ | Benzonitrile | 7 |
| 3 | $RuCl_3$•n $H_2O$ | DMF | trace |
| **4** | **$RuCl_3$•n $H_2O$** | **Dimethylaminoethanol** | **15** |
| 5 | $RuCl_3$ (dried) | Dimethylaminoethanol | 15 |
| 6 | $RuCl_2(DMSO)_4$ | Dimethylaminoethanol | 14 |

[a] Isolated yield.

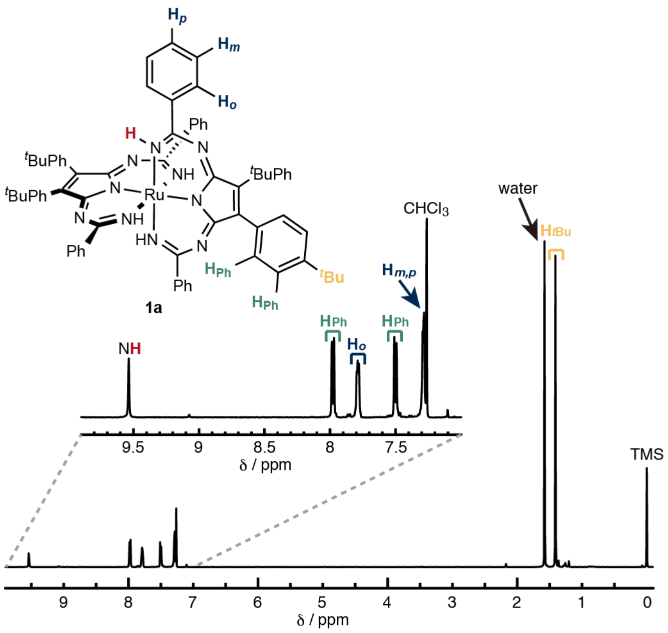


**Supplementary Fig. 1** $^1$H NMR spectra and peak assignment of **1a** in $CDCl_3$.

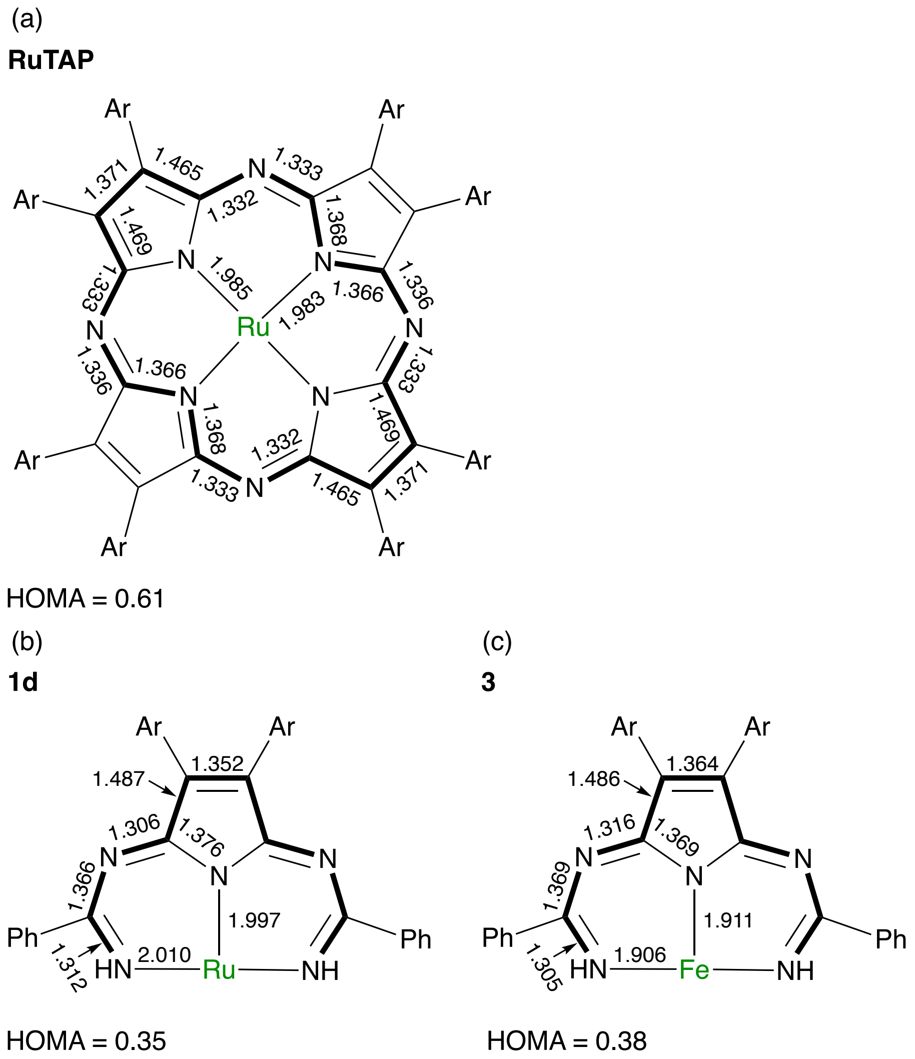


**Supplementary Fig. 2** Selected bond distances (Å) and HOMA (harmonic oscillator model of aromaticity) values[iv] of the X-ray structure of (a) **RuTAP**, (b) **1d** and (c) **3**. The bold lines indicate bonds for HOMA calculations.

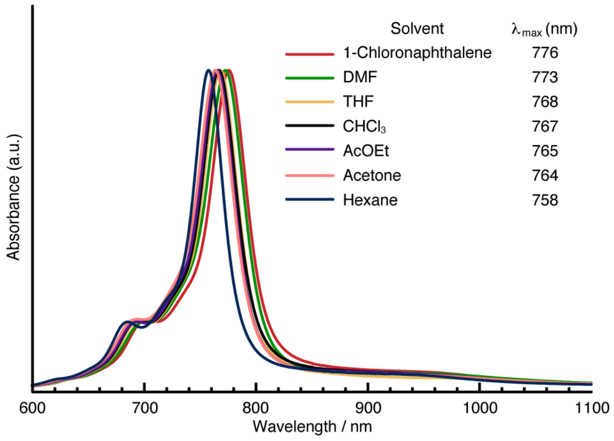


**Supplementary Fig. 3** UV–vis–NIR absorption spectra of **1a** in various solvents.

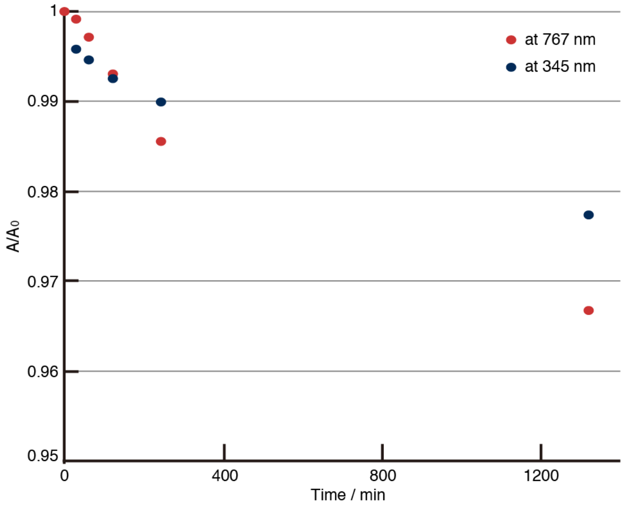


**Supplementary Fig. 4** Time-dependent optical density of **1a** at the absorption maximum wavelength upon irradiation. The original optical density before irradiation was normalized at the absorption maximum. Solution of compound in air-saturated $CHCl_3$ (ca. 2.5 x $10^{-5}$ M) was irradiated using a halogen lamp (5,000 lx).

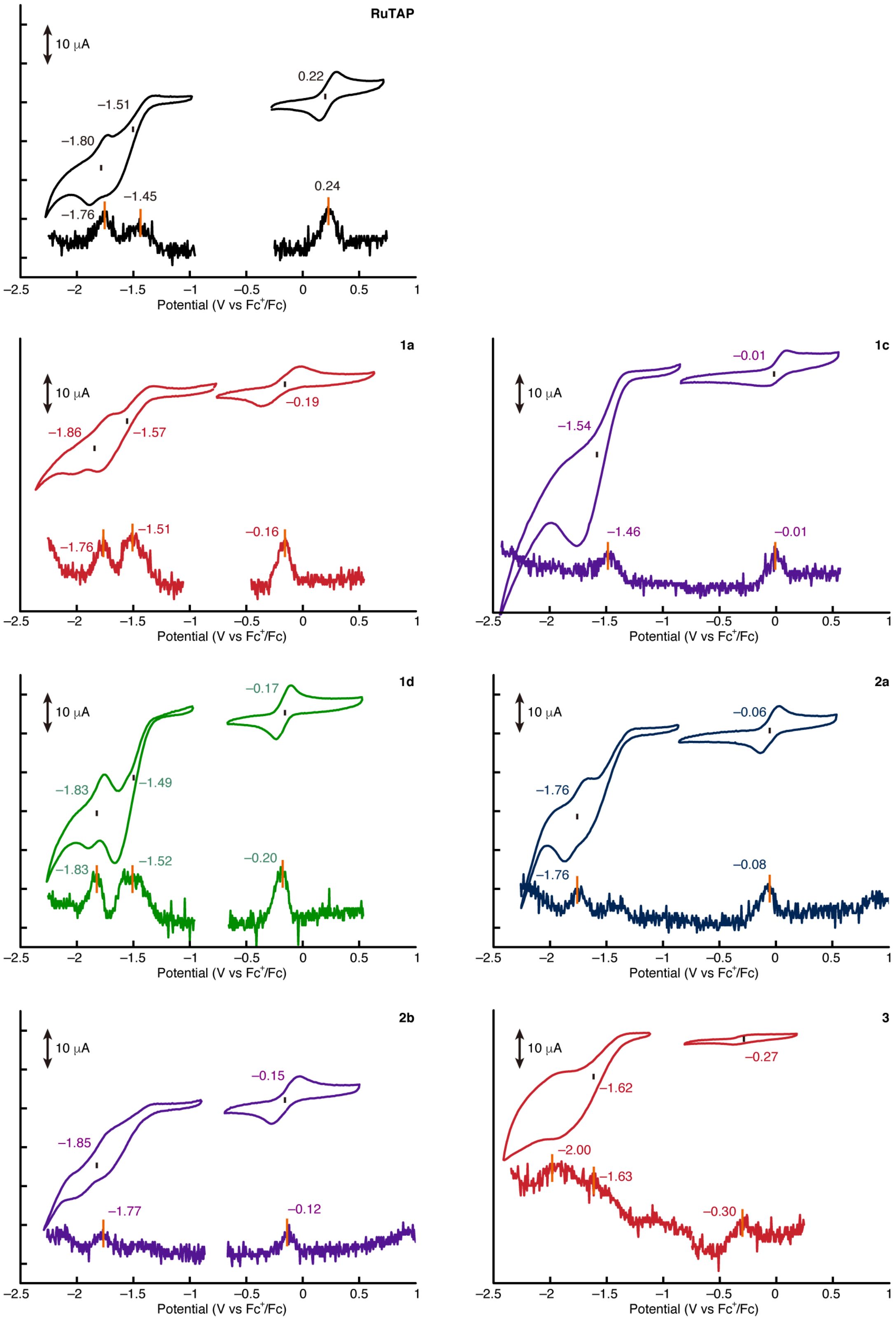


**Supplementary Fig. 5** Cyclic voltammograms (CVs) and differential pulse voltammograms (DPVs) of **RuTAP**, **1a**, **1c**, **1d, 2a**, **2b**, and **3**; [analyte] = 0.5 mM;

solvent: THF; supporting electrolyte: 0.1 M [$n$Bu$_4$N][ClO$_4$]. All potentials are referenced to the ferrocene/ferrocenium couple.

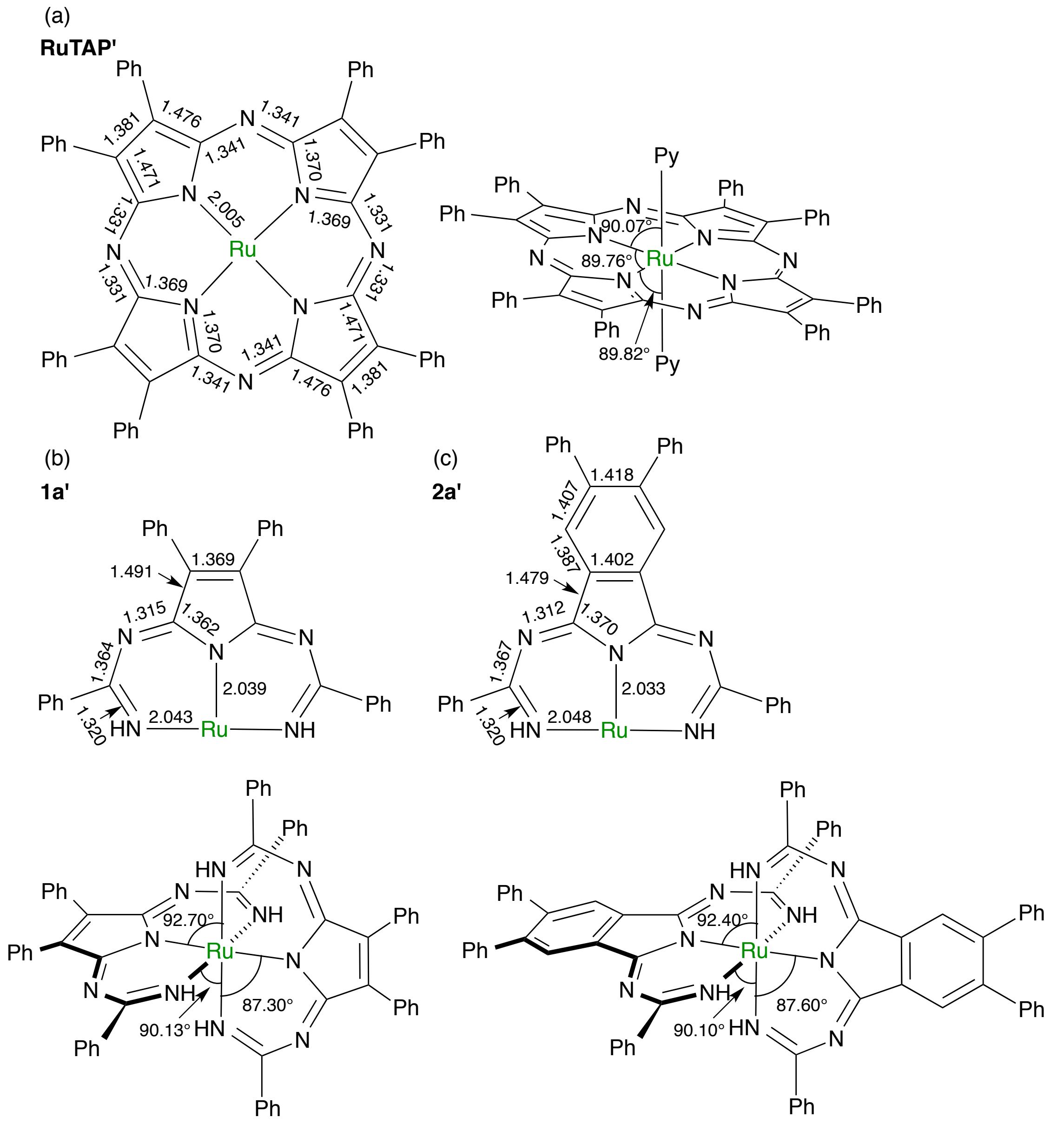


**Supplementary Fig. 6** Selected bond distances (Å) and angles (degree) of the calculated optimized structure of (a) **RuTAP’**, (b) **1a’** and (c) **2a’**.

**Supplementary Table 5.** Calculated excitation wavelengths (λ) and oscillator strengths (*f*) for components of selected transition energies.

| Compound | λ (nm) | *f* | Composition (%) |
|---|---|---|---|
| **RuTAP'** | 515.1 | 0.49 | HOMO → LUMO+1 (89%), HOMO–8 → LUMO (7%) |
| | 511.8 | 0.54 | HOMO → LUMO (89%), HOMO–8 → LUMO+1 (7%) |
| **1a'** | 632.8 | 0.35 | HOMO → LUMO (97%) |
| **1c'** | 607.3 | 0.36 | HOMO → LUMO (97%) |
| **2a'** | 586.7 | 0.42 | HOMO → LUMO (97%) |

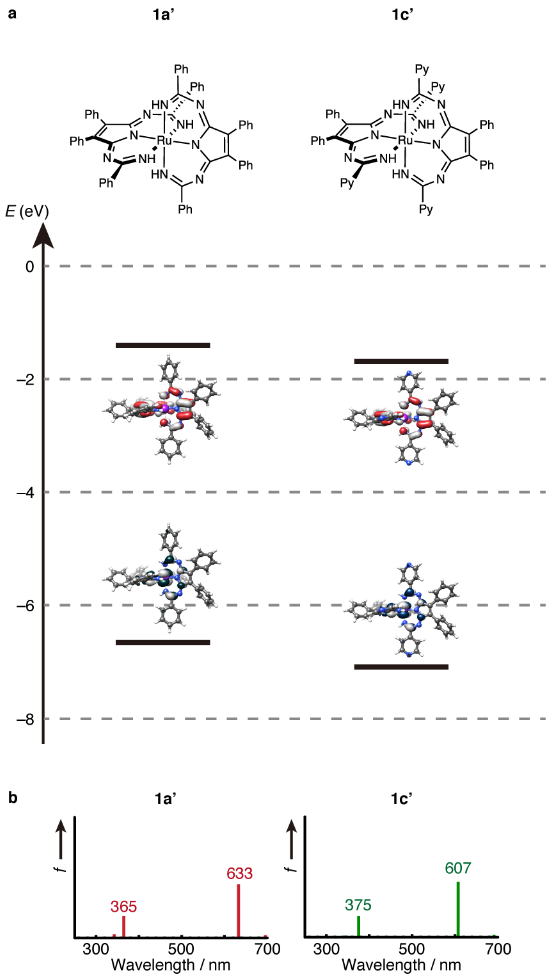


**Supplementary Fig. 7** a) Partial molecular energy diagram and orbitals of **1a'**, and **1c'** as well as b) their calculated absorption spectra. Blue and red plots indicate occupied and unoccupied MOs, respectively. The MO isovalues are 0.030. Calculations were carried out at the ωB97XD/631SDD//B3LYP/631SDD level of theory, using the polarizable continuum model (PCM) that mimicked the solvation effect of $CHCl_3$.

## Full Experimental Procedures

### Materials

Unless otherwise noted, materials were purchased from Tokyo Kasei Co., Aldrich Inc., and other commercial suppliers and were used after appropriate purification (distillation or recrystallization).

Diimine **5a–c** and **6a** were synthesized according to published procedures.[v]

### Synthesis of 1,3-diiminoisoindoline derivative 6b

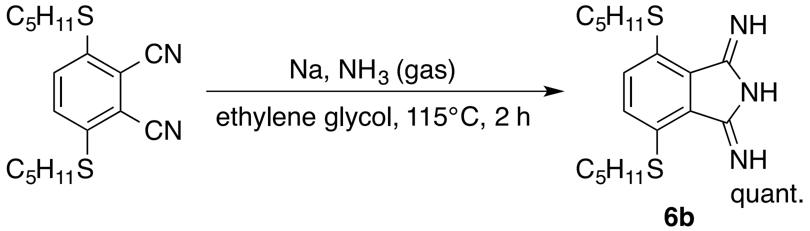


**6b**

3, 6-Bis(pentylthio)-phthalonitrile[vi] (3.0 g, 9 mmol) was suspended in ethylene glycol (40 mL) and heated to 115°C. Sodium (ca. 10 mg) was added to the solution and gaseous $NH_3$ was bubbled through the suspension for 2 h. Then the reaction mixture was poured into iced water (350 mL). After the mixture was stirred for 30 min at rt, the precipitate was collected by filteration and washed with water repeatedly. The target compound with some impurities was obtained as a dark yellow powder (3.2 g, 9 mmol, quant yield), and used for the next reaction without further purification. $^1$H NMR (500 MHz, $CDCl_3$, 1 mM): $\delta$ 7.42 (brs, 3H), 2.95 (brs, 4H), 1.54 (brs, 4H), 1.32 (brs, 8H), 0.87 (t, 6H, $J$ = 7.1 Hz); HR-MS (ESI-MS): $m/z$ = 350.1718, calcd for $(C_{18}H_{28}N_3S_2)^+$ = 350.1719 $[(M+H)^+]$.

**Synthesis of RuTAP**

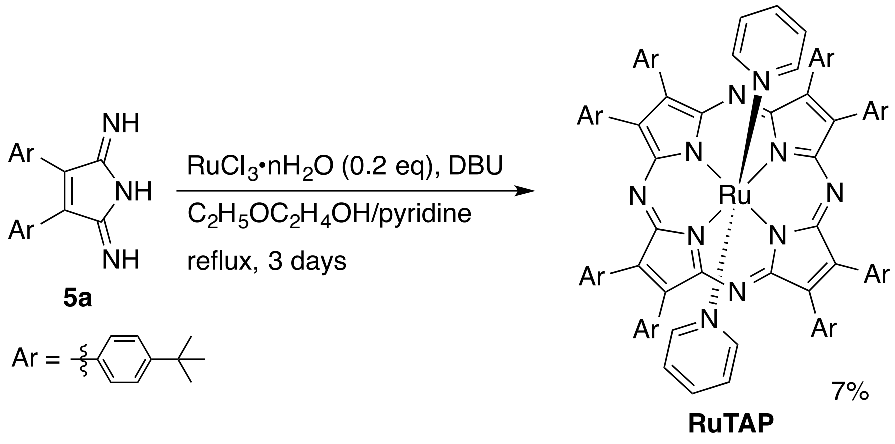


Under Ar atmosphere, diimine **5a** (360 mg, 1.00 mmol), ruthenium trichloride (45.7 mg, 0.22 mmol) and DBU (1 mL, 6.7 mmol) were dissolved in pyridine (1.5 mL) and 2-ethoxyethanol (5 mL). After the mixture was stirred under reflux for 3 days, the reaction mixture was concentrated. The residue was dissolved in water/methanol (1/1 v/v) mixture and insoluble part was collected by filtration. The black powder was purified by silica gel column chromatography ($CHCl_3$/Hexane = 3/1 v/v). The title compound was obtained as a dark red solid (29.8 mg, 18.3 μmol, 7%). $^1H$ NMR (500 MHz, $CDCl_3$, 1 mM): $\delta$ 8.26 (d, 16H, $J$ = 8.0 Hz, $^t$BuPh-Ph$H$), 7.55 (d, 16H, $J$ = 8.0 Hz, $^t$BuPh-Ph$H$), 6.27 (t, 2H, $J$ = 7.5 Hz, *p*-pyridyl), 5.52 (t, 4H, $J$ = 6.9 Hz, *m*-pyridyl), 3.03 (t, 4H, $J$ = 5.2 Hz, *o*-pyridyl), 1.48 (s, 72H, $^t$BuPh-$^t$Bu$H$); Ultraviolet-Vis-NIR ($CHCl_3$): $\lambda_{max}$ ($\varepsilon$($M^{-1}cm^{-1}$)) 598 (89,000), 550 (30,000), 480 (30,000), 353 (74,000), 285 (78,000); HR-MS (MALDI-MS): $m/z$ = 1628.8283, calcd for $(C_{106}H_{114}N_{10}Ru)^+$ = 1628.8295 [$(M)^+$].

**Synthesis of 1a**

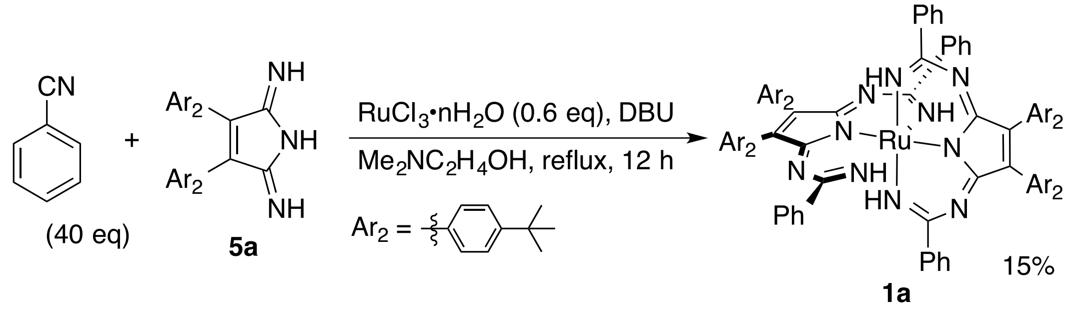


Under an Ar atmosphere, diimine **5a** (89.3 mg, 0.25 mmol) and ruthenium trichloride (31.5 mg, 0.15 mmol) were dissolved in benzonitrile (1.0 mL, 9.7 mmol) and dimethylaminoethanol (0.5 mL), followed by the addition of DBU (0.1 mL, 0.67 mmol). The mixture was stirred under reflux for 12 h, before it was concentrated. The residue was dissolved and extracted with $CHCl_3$. The organic layer was washed with brine and dried over $Na_2SO_4$, filtered and concentrated *in vacuo*. The product was purified by column chromatography on silica gel (eluent: $CHCl_3$/Hexane = 1/1 v/v) to provide **1a** as a moss-green solid (23.6 mg, 18.8 µmol, 15%) after recrystallization from methanol. $^1H$ NMR (500 MHz, $CDCl_3$, 1 mM): $\delta$ 9.53 (s, 4H, imine-N*H*), 7.98 (d, 8H, *J* = 8.5 Hz, pyrrole-$^t$BuPh-Ph*H*), 7.80–7.78 (m, 8H, ArCN-Ph*H*), 7.50 (d, 8H, *J* = 8.5 Hz, pyrrole-$^t$BuPh-Ph*H*), 7.29–7.26 (m, 12H, ArCN-Ph*H*), 1.41 (s, 36H, pyrrole-$^t$BuPh-$^t$Bu*H*); Ultraviolet-Vis-NIR ($CHCl_3$): $\lambda_{max}$ ($\varepsilon$($M^{-1}cm^{-1}$)) 767 (35,000), 345 (63,000); HR-MS (MALDI-MS): *m*/*z* = 1230.5290, calcd. for $(C_{76}H_{76}N_{10}Ru)^+$ = 1230.5313 $[(M)^+]$.

**Synthesis of 1b**

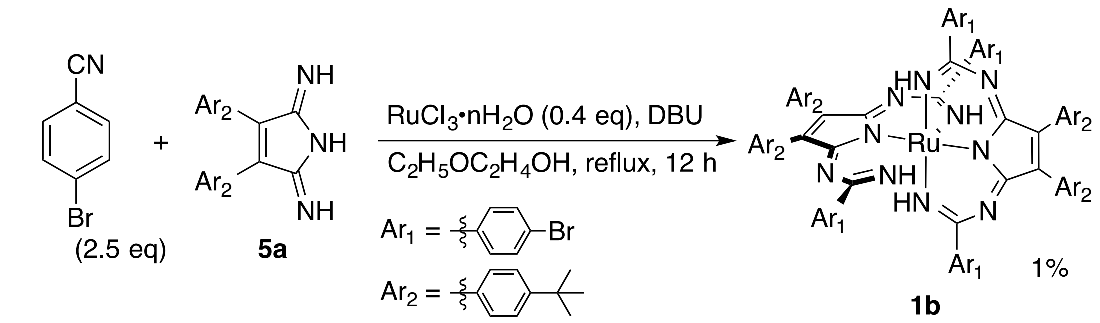


Under Ar atmosphere, diimine **5a** (90.2 mg, 0.25 mmol), ruthenium trichloride (22.5 mg, 0.11 mmol), and 4-bromobenzonitrile (109 mg, 0.60 mmol) were dissolved in 2-ethoxyethanol (0.5 mL), then DBU (0.1 mL, 0.67 mmol) was added. After the mixture was stirred under reflux for 12 h, the reaction mixture was concentrated. The residue was dissolved and extracted with $CHCl_3$. The organic layer was washed with brine and dried over $Na_2SO_4$, filtered and concentrated *in vacuo*. The product was purified by silica gel column chromatography ($CHCl_3$/Hexane = 2/1 v/v). The title compound was obtained as a yellowish-green solid (2.1 mg, 1.4 μmol, 1%) after recrystallization from methanol. $^1$H NMR (500 MHz, $CDCl_3$, 1 mM): $\delta$ 9.44 (s, 4H, imine-N*H*), 7.93 (d, 8H, $J$ = 8.6 Hz, pyrrole-$^t$BuPh-Ph*H*), 7.65 (d, 8H, $J$ = 8.6 Hz, $Ar_1$CN-Ph*H*), 7.50 (d, 8H, $J$ = 8.6 Hz, pyrrole-$^t$BuPh-Ph*H*), 7.42 (d, 8H, $J$ = 9.2 Hz, $Ar_1$CN-Ph*H*), 1.41 (s, 36H, pyrrole-$^t$BuPh-$^t$Bu*H*); Ultraviolet-Vis-NIR ($CHCl_3$): $\lambda_{max}$ ($\varepsilon$($M^{-1}cm^{-1}$)) 764 (23,000), 693 (5,000), 350 (46,000) nm; HR-MS (MALDI-MS): *m/z* = 1542.1714, calcd for $(C_{76}H_{72}Br_4N_{10}Ru)^+$ = 1542.1713 [$(M)^+$].

**Synthesis of 1c**

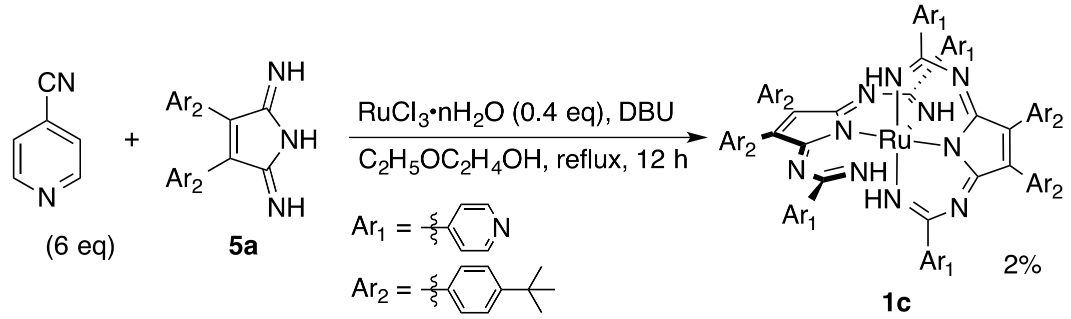


Under Ar atmosphere, diimine **5a** (180.3 mg, 0.50 mmol) and ruthenium trichloride (31.3 mg, 0.21 mmol) and 4-cyanopyridine (332.8 mg, 3.2 mmol) were dissolved in 2-ethoxyethanol (1.0 mL), then DBU (0.2 mL, 1.3 mmol) was added. After the mixture was stirred under reflux for 12 h, the reaction mixture was concentrated. The residue was dissolved in water/methanol (1/1 v/v) mixture and insoluble part was collected by filtration. The black powder was purified by silica gel column chromatography ($CHCl_3$/Methanol = 100/1 v/v). The title compound was obtained as a green solid (7.2 mg, 5.8 μmol, 2%) after recrystallization from methanol. $^1$H NMR (500 MHz, $CDCl_3$, 1 mM): $\delta$ 9.62 (s, 4H, imine-N*H*), 8.59 (d, 8H, $J$ = 5.2 Hz, pyridyl-*H*), 7.95 (d, 8H, $J$ = 8.6 Hz, pyrrole-$^t$BuPh-Ph*H*), 7.66 (d, 8H, $J$ = 5.8 Hz, pyridyl-*H*), 7.55 (d, 8H, $J$ = 8.1 Hz, pyrrole-$^t$BuPh-Ph*H*), 1.42 (s, 36H, pyrrole-$^t$BuPh-$^t$Bu*H*); Ultraviolet-Vis-NIR ($CHCl_3$): $\lambda_{max}$ ($\varepsilon$($M^{-1}cm^{-1}$)) 732 (23,000), 664 (5,000), 350 (31,000) nm; HR-MS (MALDI-MS): $m/z$ = 1234.5121, calcd for $(C_{72}H_{72}N_{14}Ru)^+$ = 1234.5122 [$(M)^+$].

**Synthesis of 1d**

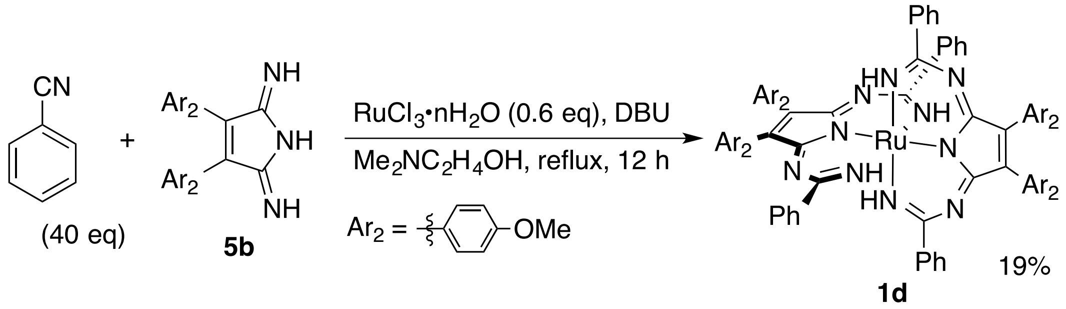


Under Ar atmosphere, diimine **5b** (77.4 mg, 0.25 mmol) and ruthenium trichloride (30.6 mg, 0.15 mmol) were dissolved in benzonitrile (1.0 mL, 9.7 mmol) and dimethylaminoethanol (0.5 mL), then DBU (0.1 mL, 0.67 mmol) was added. After the mixture was stirred under reflux for 12 h, the reaction mixture was concentrated. The residue was dissolved and extracted with $CHCl_3$. The organic layer was washed with brine and dried over $Na_2SO_4$, filtered and concentrated *in vacuo*. The product was purified by silica gel column chromatography ($CHCl_3$/Hexane = 4/1 v/v). The title compound was obtained as a green solid (26.2 mg, 23.3 μmol, 19%) after recrystallization from methanol. $^1$H NMR (500 MHz, $CDCl_3$, 1 mM): $\delta$ 9.53 (s, 4H, imine-N*H*), 7.96 (d, 8H, *J* = 8.6 Hz, pyrrole-MeOPh-Ph*H*), 7.79-7.77 (m, 8H, ArCN-Ph*H*), 7.29-7.26 (m, 12H, ArCN-Ph*H*), 7.04 (d, 8H, *J* = 8.6 Hz, pyrrole-MeOPh-Ph*H*), 3.92 (s, 12H, pyrrole-MeOPh-Me*H*); Ultraviolet-Vis-NIR ($CHCl_3$): $\lambda_{max}$ ($\varepsilon$($M^{-1}cm^{-1}$)) 766 (40,000), 693 (8,800), 348 (66,000) nm; *Anal.* C, 67.27 H, 4.49 N, 12.26, calcd for ($C_{64}H_{54}N_{10}O_5Ru$) = C, 66.95; H, 4.74; N, 12.20 (**1d** • $H_2O$). HR-MS (MALDI-MS): *m/z* = 1126.3210, calcd for $(C_{64}H_{52}N_{10}O_4Ru)^+$ = 1126.3216 [$(M)^+$].

**Synthesis of 1e**

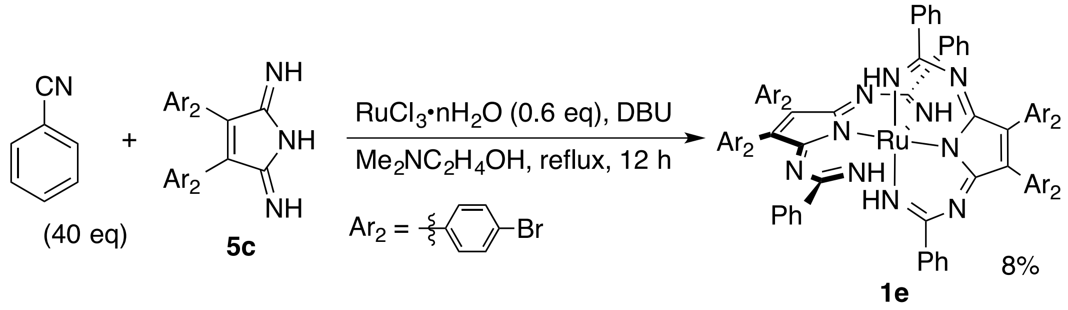


Under Ar atmosphere, diimine **5c** (100.5 mg, 0.25 mmol) and ruthenium trichloride (31.6 mg, 0.15 mmol) were dissolved in benzonitrile (1.0 mL, 9.7 mmol) and dimethylaminoethanol (0.5 mL), then DBU (0.1 mL, 0.67 mmol) was added. After the mixture was stirred under reflux for 12 h, the reaction mixture was concentrated. The residue was dissolved and extracted with $CHCl_3$. The organic layer was washed with brine and dried over $Na_2SO_4$, filtered and concentrated *in vacuo*. The product was purified by silica gel column chromatography ($CHCl_3$). The title compound was obtained as a green solid (13.4 mg, 10.1 μmol, 8%) after recrystallization from methanol. $^1$H NMR (500 MHz, $CDCl_3$, 1 mM): $\delta$ 9.55 (s, 4H, imine-N*H*), 7.84 (d, 8H, *J* = 8.6 Hz, pyrrole-BrPh-Ph*H*), 7.73-7.72 (m, 8H, ArCN-Ph*H*), 7.64 (d, 8H, *J* = 8.6 Hz, pyrrole-BrPh-Ph*H*), 7.32-7.31 (m, 12H, ArCN-Ph*H*); Ultraviolet-Vis-NIR ($CHCl_3$): $\lambda_{max}$ ($\varepsilon$($M^{-1}cm^{-1}$)) 777 (27,000), 704 (8,100), 344 (62,000) nm; HR-MS (MALDI-MS): *m/z* = 1317.9211, calcd for $(C_{60}H_{40}Br_4N_{10}Ru)^+$ = 1317.9210 [$(M)^+$].

**Synthesis of 2a**

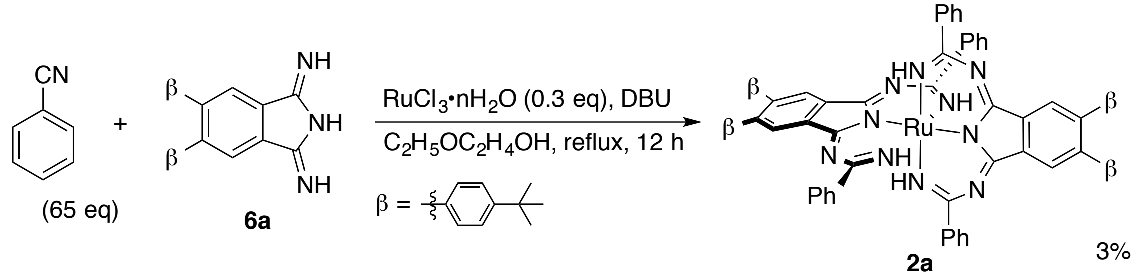


Under Ar atmosphere, diimine **6a** (304.5 mg, 0.74 mmol) and ruthenium trichloride (45.5 mg, 0.22 mmol) were dissolved in benzonitrile (0.5 mL, 4.9 mmol) and 2-ethoxyethanol (1.5 mL), then DBU (0.3 mL, 2.0 mmol) was added. After the mixture was stirred under reflux for 12 h, the reaction mixture was concentrated. The residue was dissolved in water/methanol (1/1 v/v) mixture and insoluble part was collected by filtration. The black powder was purified by silica gel column chromatography ($CHCl_3$/Hexane = 4/1 v/v). The title compound was obtained as a green solid (16.7 mg, 12.5 μmol, 3%) after recrystallization from methanol. $^1H$ NMR (500 MHz, $CDCl_3$, 1 mM): $\delta$ 9.26 (s, 4H, imine-N*H*), 8.55 (s, 4H, isoindole-α*H*), 7.85-7.84 (m, 8H, ArCN-Ph*H*), 7.34-7.26 (m, 28H, isoindole-$^t$BuPh-Ph*H* and ArCN-Ph*H*), 1.35 (s, 36H, isoindole-$^t$BuPh-$^t$Bu*H*); Ultraviolet-Vis-NIR ($CHCl_3$): $\lambda_{max}$ ($\varepsilon$($M^{-1}cm^{-1}$)) 717 (34,000), 655 (10,000), 330 (81,000), 284 (74,000) nm; HR-MS (MALDI-MS): *m/z* = 1330.5618, calcd for $(C_{84}H_{80}N_{10}Ru)^+$ = 1330.5628 [$(M)^+$].

**Synthesis of 2b**

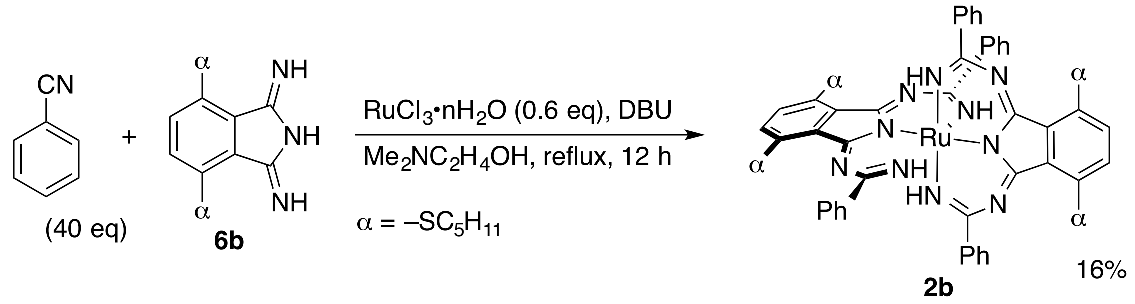


Under Ar atmosphere, diimine **6b** (174.8 mg, 0.50 mmol) and ruthenium trichloride (62.2 mg, 0.3 mmol) were dissolved in benzonitrile (2.0 mL, 20 mmol) and dimethylaminoethanol (1 mL), then DBU (0.22 mL, 1.5 mmol) was added. After the mixture was stirred under reflux for 12 h, the reaction mixture was concentrated. The residue was dissolved and extracted with $CHCl_3$. The organic layer was washed with brine and dried over $Na_2SO_4$, filtered and concentrated *in vacuo*. The product was purified by silica gel column chromatography ($CHCl_3$). The title compound was obtained as a green solid (48.4 mg, 40.0 μmol, 16%) after recrystallization from methanol. $^1$H NMR (500 MHz, $CDCl_3$, 1 mM): $\delta$ 9.26 (s, 4H, imine-N*H*), 8.01-7.99 (m, 8H, ArCN-Ph*H*), 7.52 (s, 4H, isoindole-β*H*), 7.28-7.27 (m, 12H, ArCN-Ph*H*), 3.22 (t, 8H, $J$ = 7.5 Hz, $SC_5H_{11}$-*H*), 1.97-1.94 (m, 8H, $SC_5H_{11}$-*H*), 1.62-1.59 (m, 8H, $SC_5H_{11}$-*H*), 1.47-1.43 (m, 8H, $SC_5H_{11}$-*H*), 0.96 (t, 12H, $J$ = 7.5 Hz, $SC_5H_{11}$-*H*); Ultraviolet-Vis-NIR ($CHCl_3$): $\lambda_{max}$ ($\varepsilon$($M^{-1}cm^{-1}$)) 745 (33,000), 683 (11,000), 418 (23,000), 272 (73,000) nm; HR-MS (MALDI-MS): $m/z$ = 1210.3869, calcd for $(C_{64}H_{72}N_{10}RuS_4)^+$ = 1210.3877 [$(M)^+$].

**Synthesis of 2c**

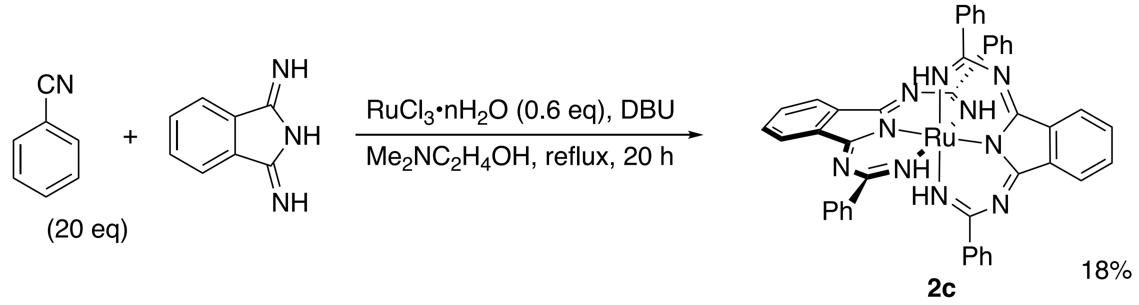


Under Ar atmosphere, 1,3-diiminoisoindoline (108.7 mg, 0.75 mmol) and ruthenium trichloride (118 mg, 0.57 mmol) were dissolved in benzonitrile (1.5 mL, 15 mmol) and dimethylaminoethanol (0.5 mL), then DBU (0.22 mL, 1.5 mmol) was added. After the mixture was stirred under reflux for 20 h, the reaction mixture was concentrated. The residue was dissolved and extracted with $CHCl_3$. The organic layer was washed with brine and dried over $Na_2SO_4$, filtered and concentrated *in vacuo*. The product was purified by silica gel column chromatography ($CHCl_3$). The title compound was obtained as a green solid (53.1 mg, 66.0 μmol, 18%) after recrystallization from methanol. $^1$H NMR (500 MHz, $CDCl_3$, 1 mM): $\delta$ 9.22 (s, 4H, imine-N*H*), 8.51-8.49 (m, 4H, isoindole-α*H*), 7.84-7.82 (m, 8H, ArCN-Ph*H*), 7.79-7.77 (m, 4H, isoindole-β*H*), 7.33-7.31 (m, 12H, ArCN-Ph*H*); Ultraviolet-Vis-NIR ($CHCl_3$): $\lambda_{max}$ ($\varepsilon$($M^{-1}cm^{-1}$)) 706 (27,000), 647 (8,600), 408 (16,000), 314 (58,000) nm; HR-MS (MALDI-MS): *m/z* = 802.1861, calcd for $(C_{44}H_{32}N_{10}Ru)^+$ = 802.1854 [$(M)^+$].

## Synthesis of 3

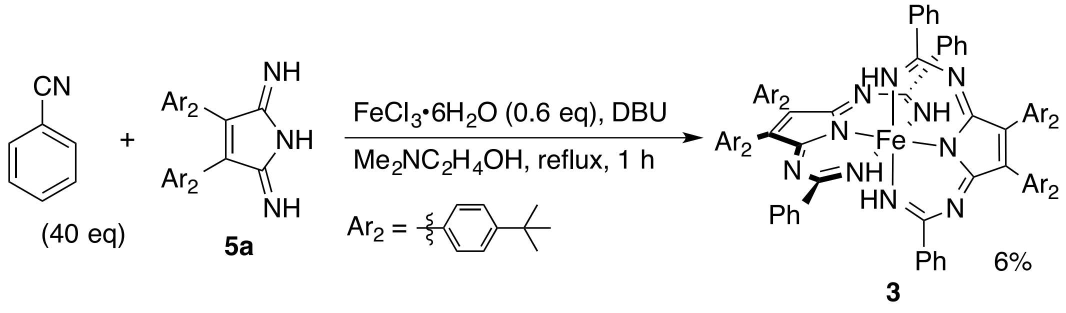


Under Ar atmosphere, diimine **5a** (91.1 mg, 0.25 mmol) and iron trichloride hexahydrate (45.0 mg, 0.17 mmol) were dissolved in benzonitrile (1.0 mL, 9.7 mmol) and dimethylaminoethanol (0.5 mL), then DBU (0.1 mL, 0.67 mmol) was added. After the mixture was stirred under reflux for 1 h, the reaction mixture was concentrated. The residue was dissolved and extracted with $CHCl_3$. The organic layer was washed with brine and dried over $Na_2SO_4$, filtered and concentrated *in vacuo*. The product was purified by silica gel column chromatography ($CHCl_3$/Hexane = 5/1 v/v). The title compound was obtained as a black solid (9.6 mg, 8.1 μmol, 6%) after recrystallization from methanol. $^1$H NMR (500 MHz, $CDCl_3$, 1 mM): $\delta$ 10.31 (s, 4H, imine-N*H*), 7.91-7.89 (m, 8H, ArCN-Ph*H*), 7.82 (d, 8H, $J$ = 8.5 Hz, pyrrole-$^t$BuPh-Ph*H*), 7.44 (d, 8H, $J$ = 8.5 Hz, pyrrole-$^t$BuPh-Ph*H*), 7.34-7.33 (m, 8H, ArCN-Ph*H*), 1.38 (s, 36H, pyrrole-$^t$BuPh-$^t$Bu*H*); Ultraviolet-Vis-NIR ($CHCl_3$): $\lambda_{max}$ ($\varepsilon$($M^{-1}cm^{-1}$)) 880 (23,000), 374 (50,000) nm; HR-MS (MALDI-MS): *m/z* = 1184.5604, calcd for $(C_{76}H_{76}N_{10}Fe)^+$ = 1184.5600 [$(M)^+$].

**Synthesis of 4**

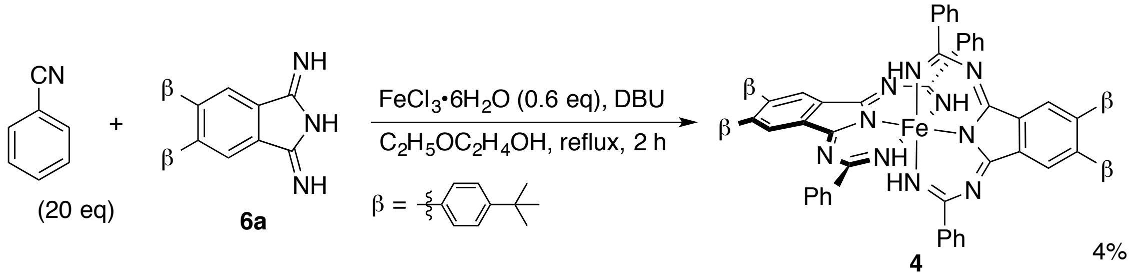


Under Ar atmosphere, diimine **6a** (295.6 mg, 0.72 mmol) and iron trichloride hexahydrate (125.1 mg, 0.46 mmol) were dissolved in benzonitrile (1.5 mL, 15 mmol) and 2-ethoxyethanol (1.5 mL), then DBU (0.3 mL, 2.0 mmol) was added. After the mixture was stirred under reflux for 2 h, the reaction mixture was concentrated. The residue was dissolved and extracted with $CHCl_3$. The organic layer was washed with brine and dried over $Na_2SO_4$, filtered and concentrated *in vacuo*. The product was purified by silica gel column chromatography ($CHCl_3$). The title compound was obtained as a brown solid (18.4 mg, 14.3 μmol, 4%) after recrystallization from methanol. $^1H$ NMR (500 MHz, $CDCl_3$, 1 mM): $\delta$ 9.90 (s, 4H, imine-N*H*), 8.44 (s, 4H, isoindole-α*H*), 7.93 (d, 8H, *J* = 6.0 Hz, isoindole-$^t$BuPh-Ph*H*), 7.36-7.23 (m, 28H, isoindole-$^t$BuPh-Ph*H* and ArCN-Ph*H*), 1.25 (s, 36H, isoindole-$^t$BuPh-$^t$Bu*H*); Ultraviolet-Vis-NIR ($CHCl_3$): $\lambda_{max}$ ($\varepsilon$($M^{-1}cm^{-1}$)) 822 (25,000), 451 (12,000), 353 (57,000), 290 (74,000) nm; HR-MS (MALDI-MS): *m/z* = 1284.5914, calcd for $(C_{84}H_{80}N_{10}Fe)^+$ = 1284.5914 [$(M)^+$].

## Copies of the NMR Spectra of Studied Compounds

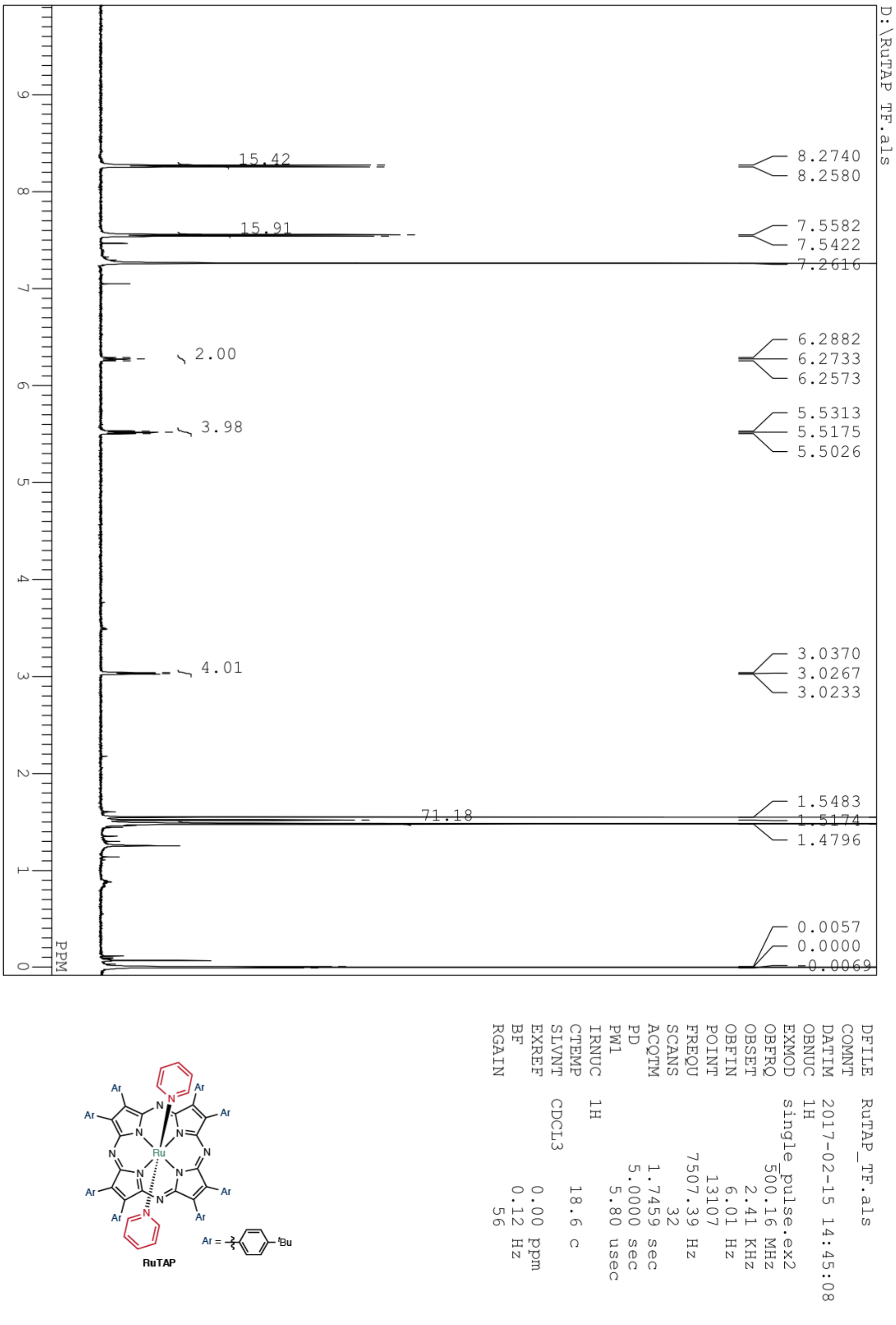


**Supplementary Fig. 8** $^{1}$H NMR spectrum of **RuTAP** in $CDCl_3$.

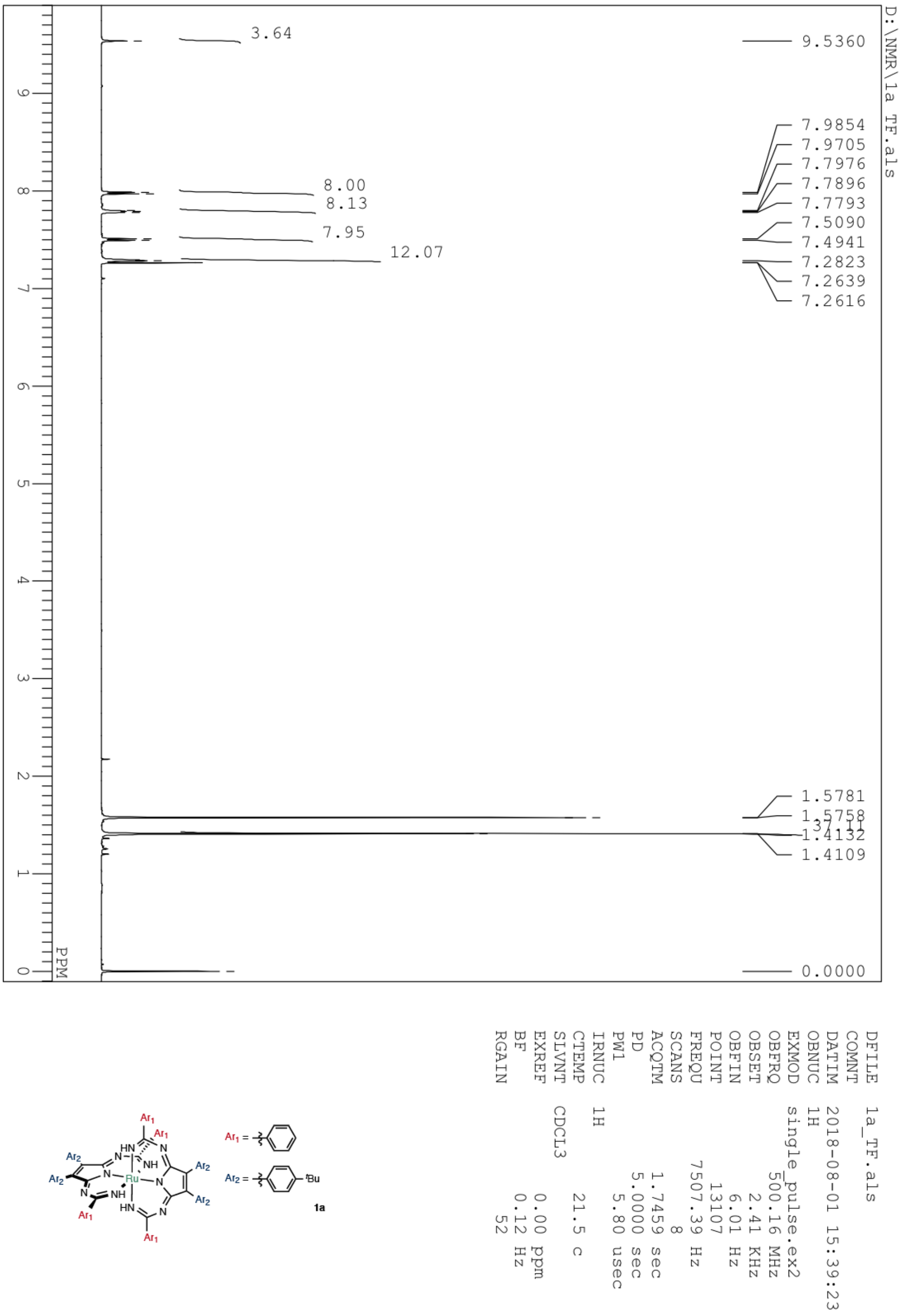


**Supplementary Fig. 9** $^{1}$H NMR spectrum of **1a** in $CDCl_3$.

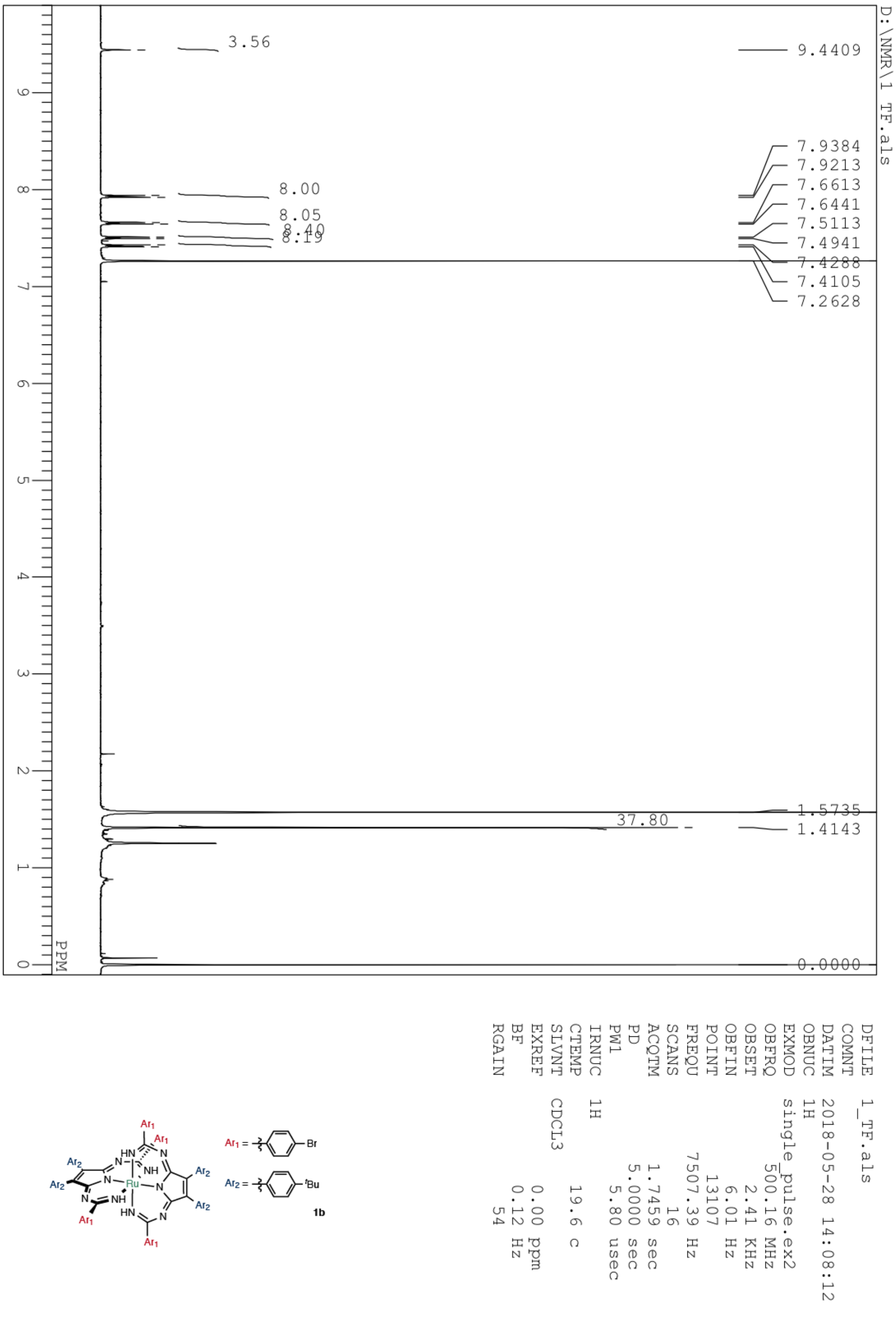


**Supplementary Fig. 10** $^{1}H$ NMR spectrum of **1b** in $CDCl_3$.

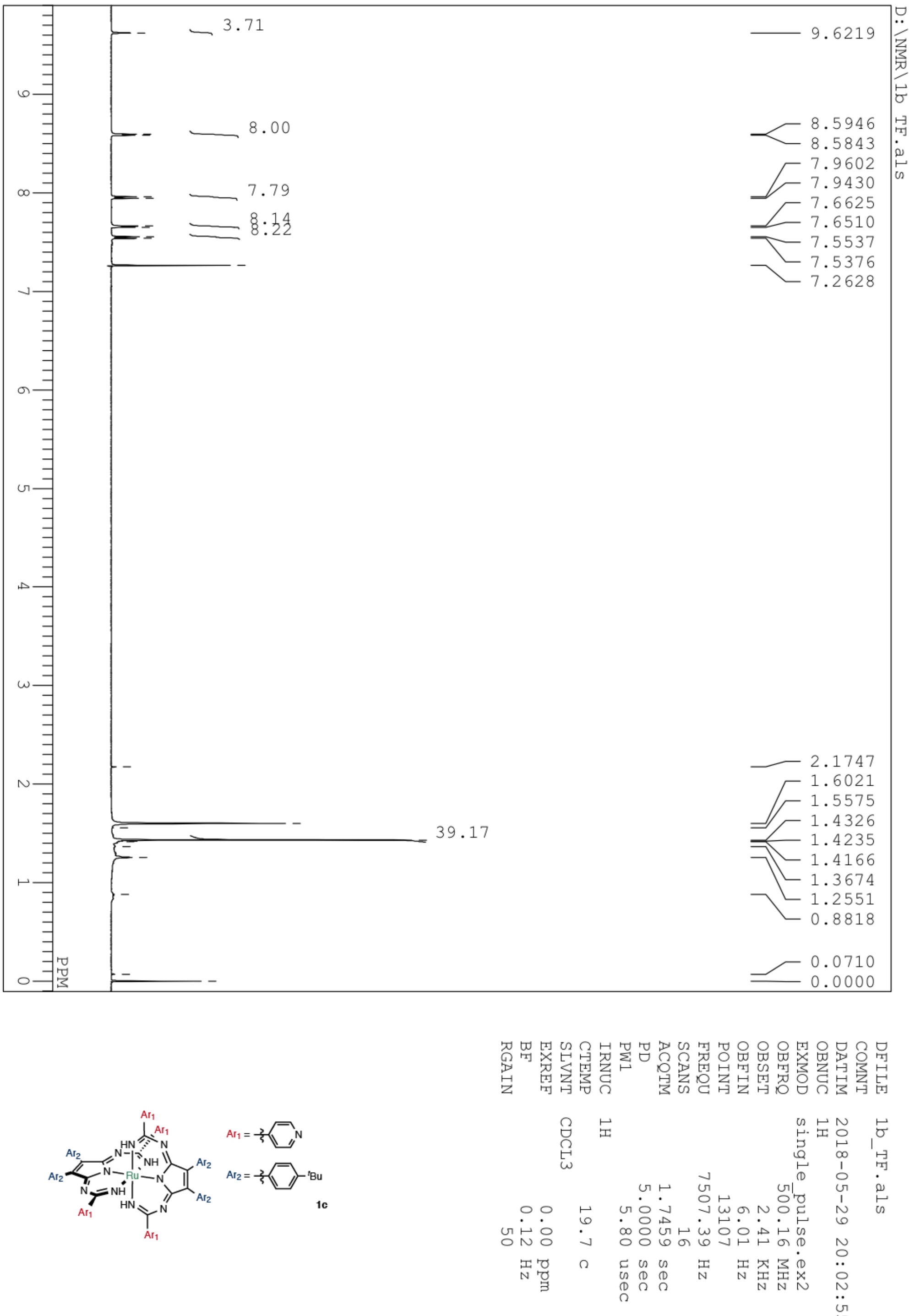


**Supplementary Fig. 11** $^1$H NMR spectrum of **1c** in $CDCl_3$.

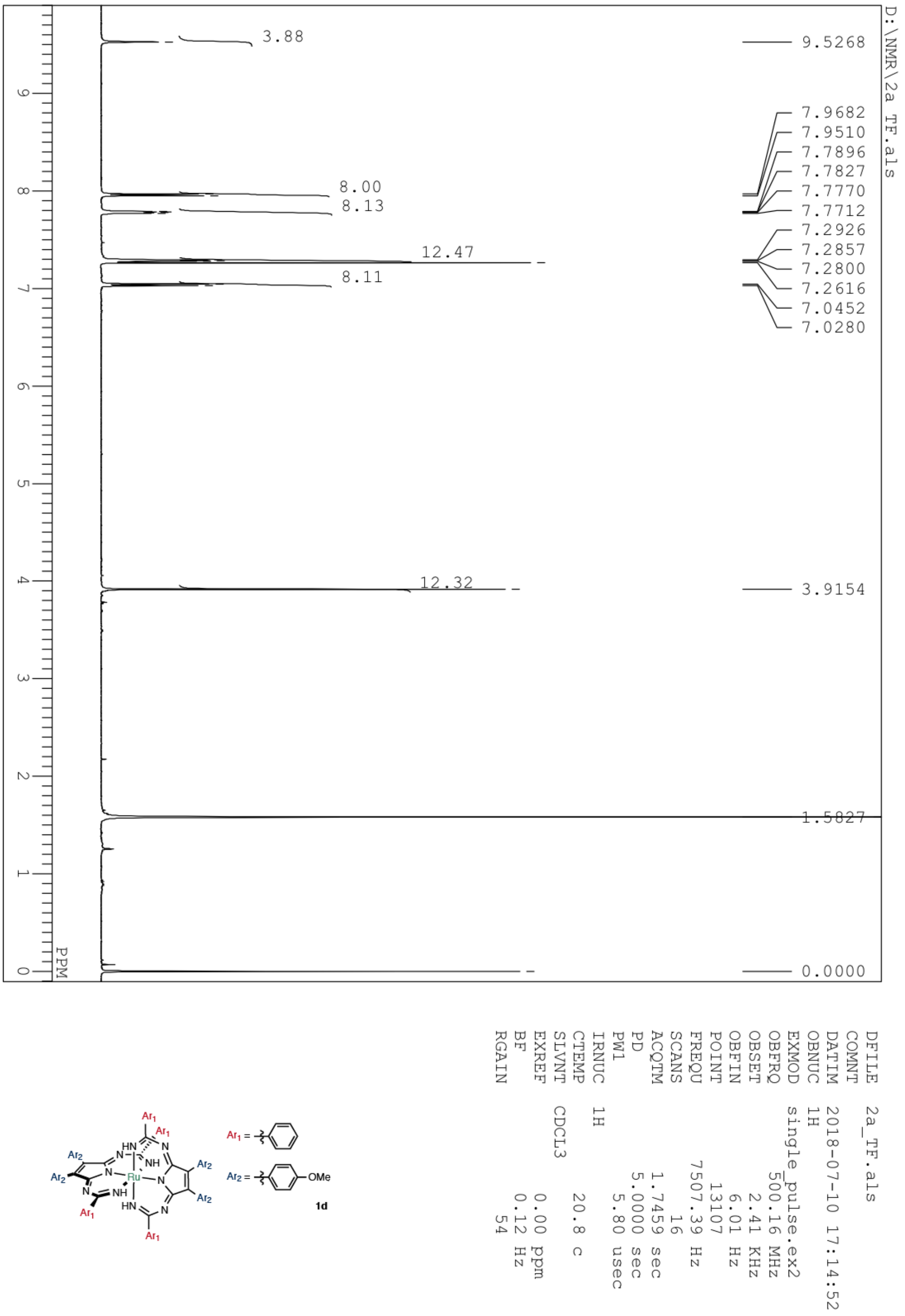


**Supplementary Fig. 12** $^{1}H$ NMR spectrum of **1d** in $CDCl_3$.

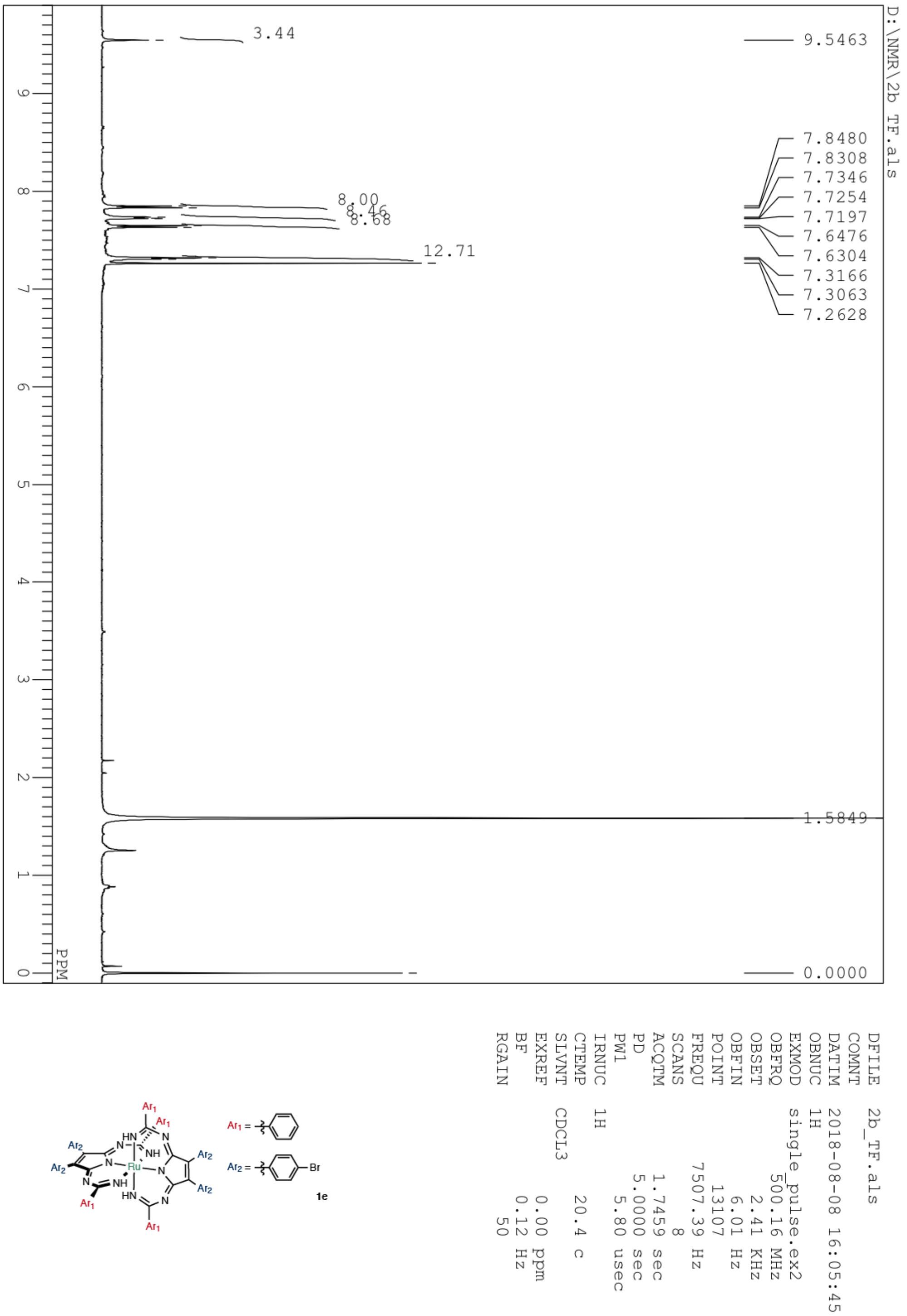


**Supplementary Fig. 13** $^{1}$H NMR spectrum of **1e** in $CDCl_3$.

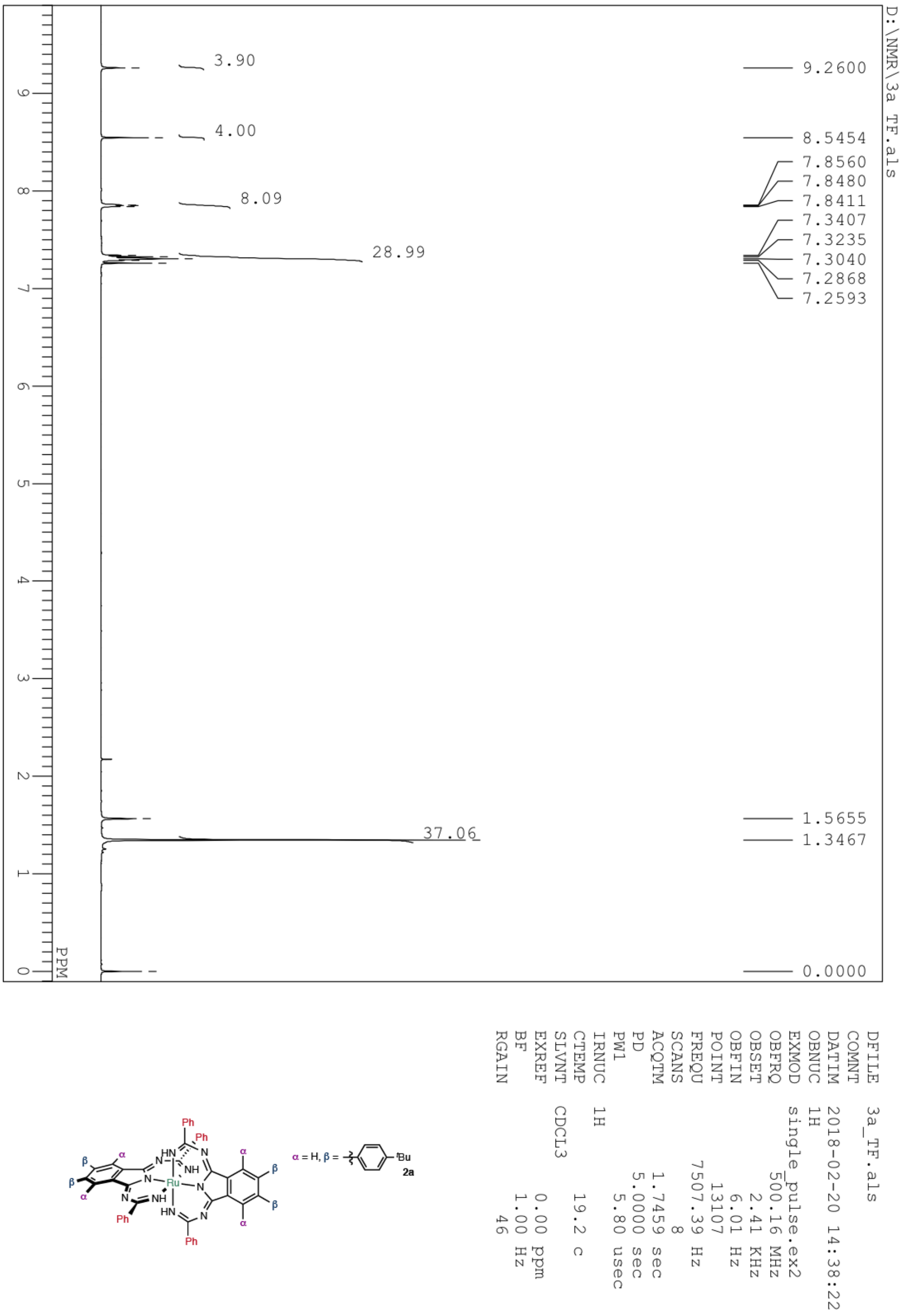


**Supplementary Fig. 14** $^{1}$H NMR spectrum of **2a** in $CDCl_3$.

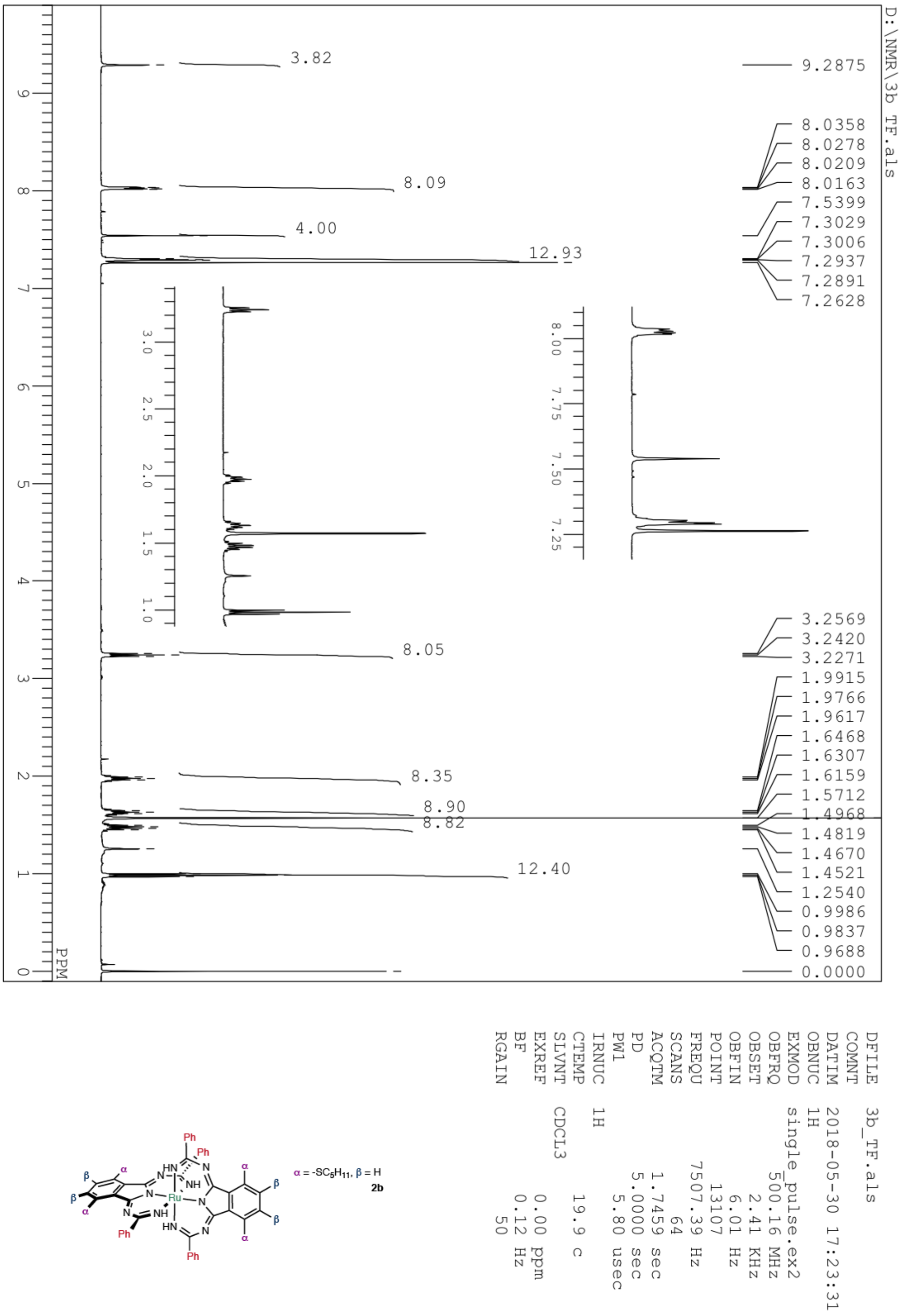


**Supplementary Fig. 15** $^{1}H$ NMR spectrum of **2b** in $CDCl_3$.

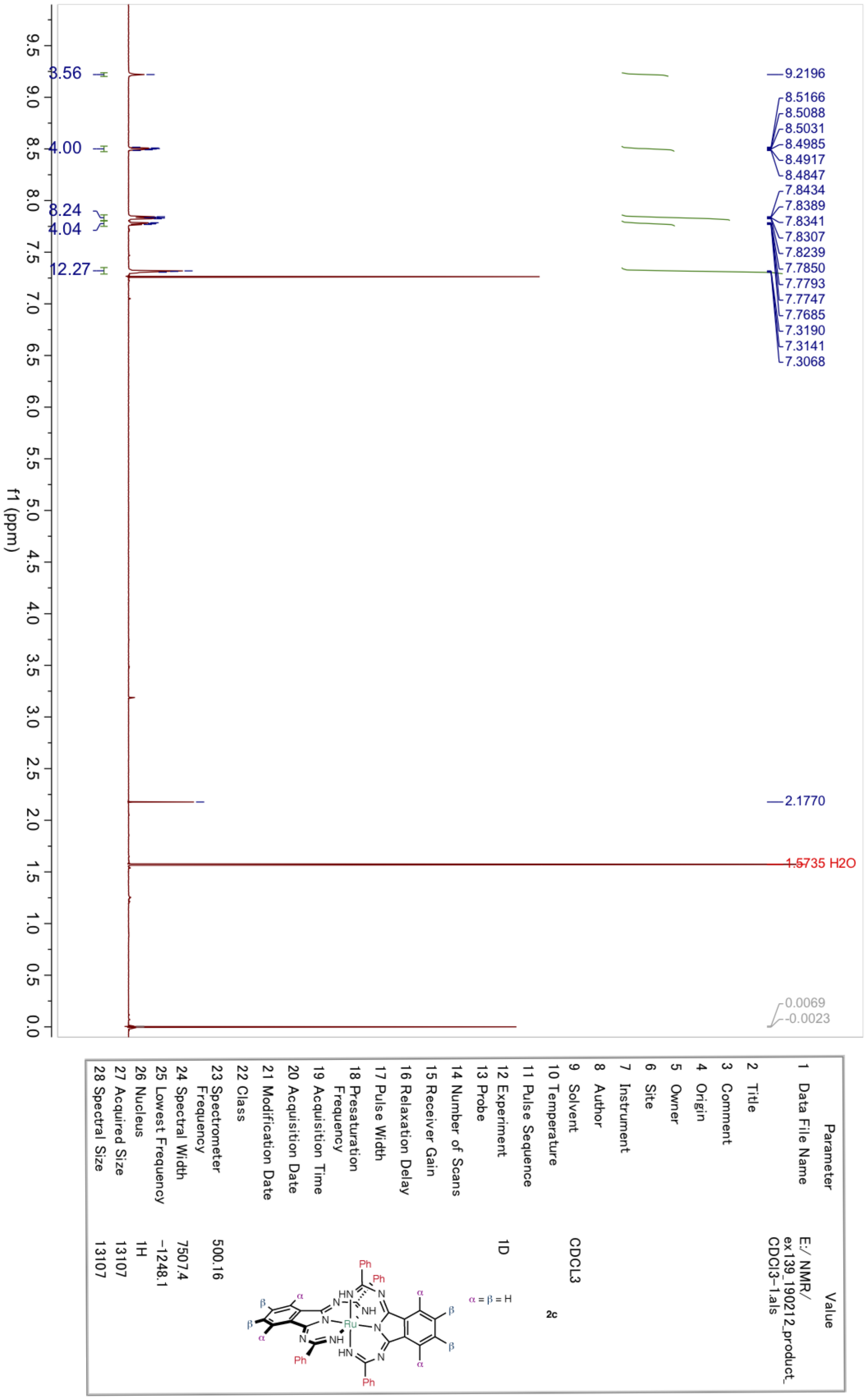


**Supplementary Fig. 16** $^{1}$H NMR spectrum of **2c** in $CDCl_3$.

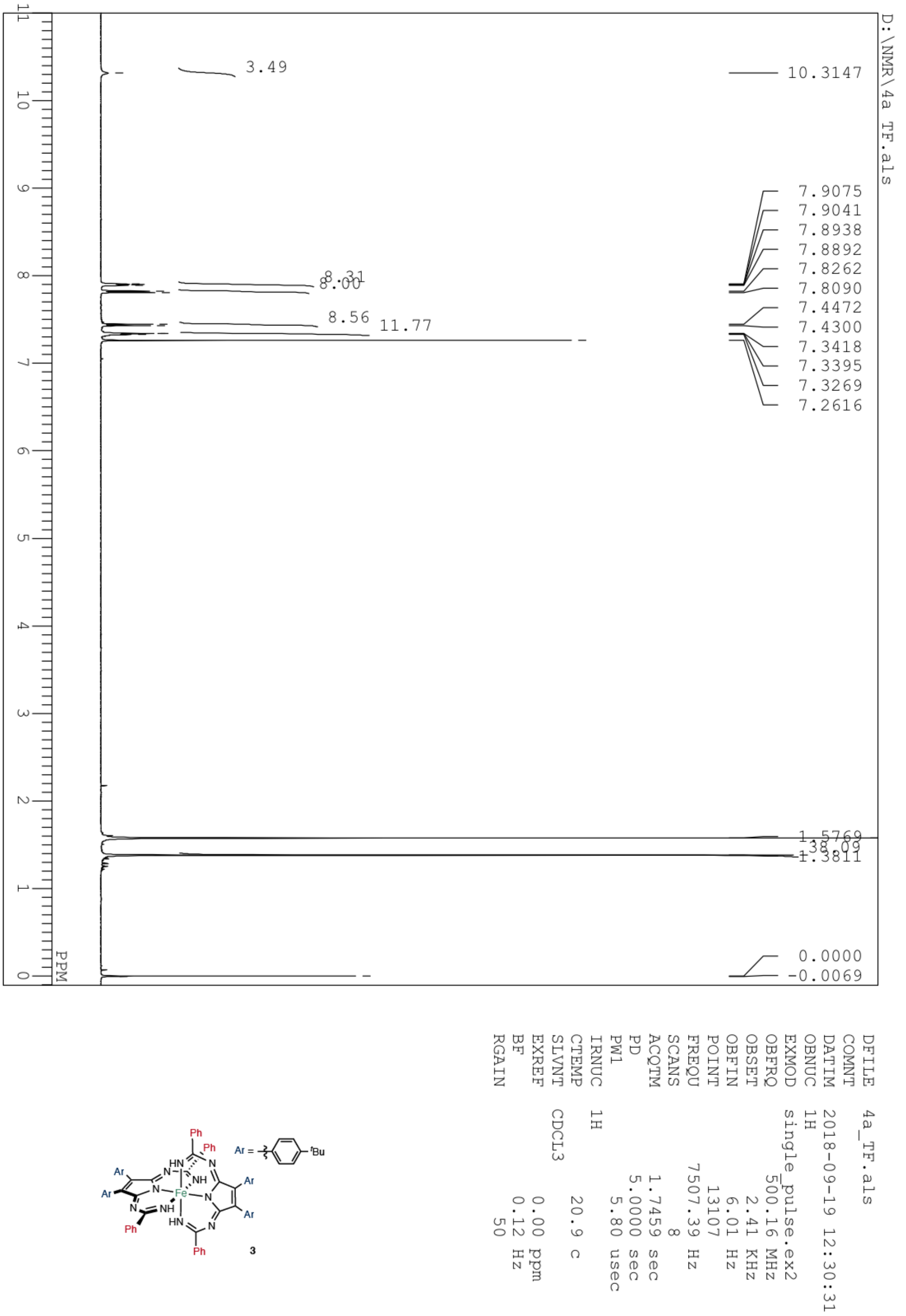


**Supplementary Fig. 17** $^{1}$H NMR spectrum of **3** in $CDCl_3$.

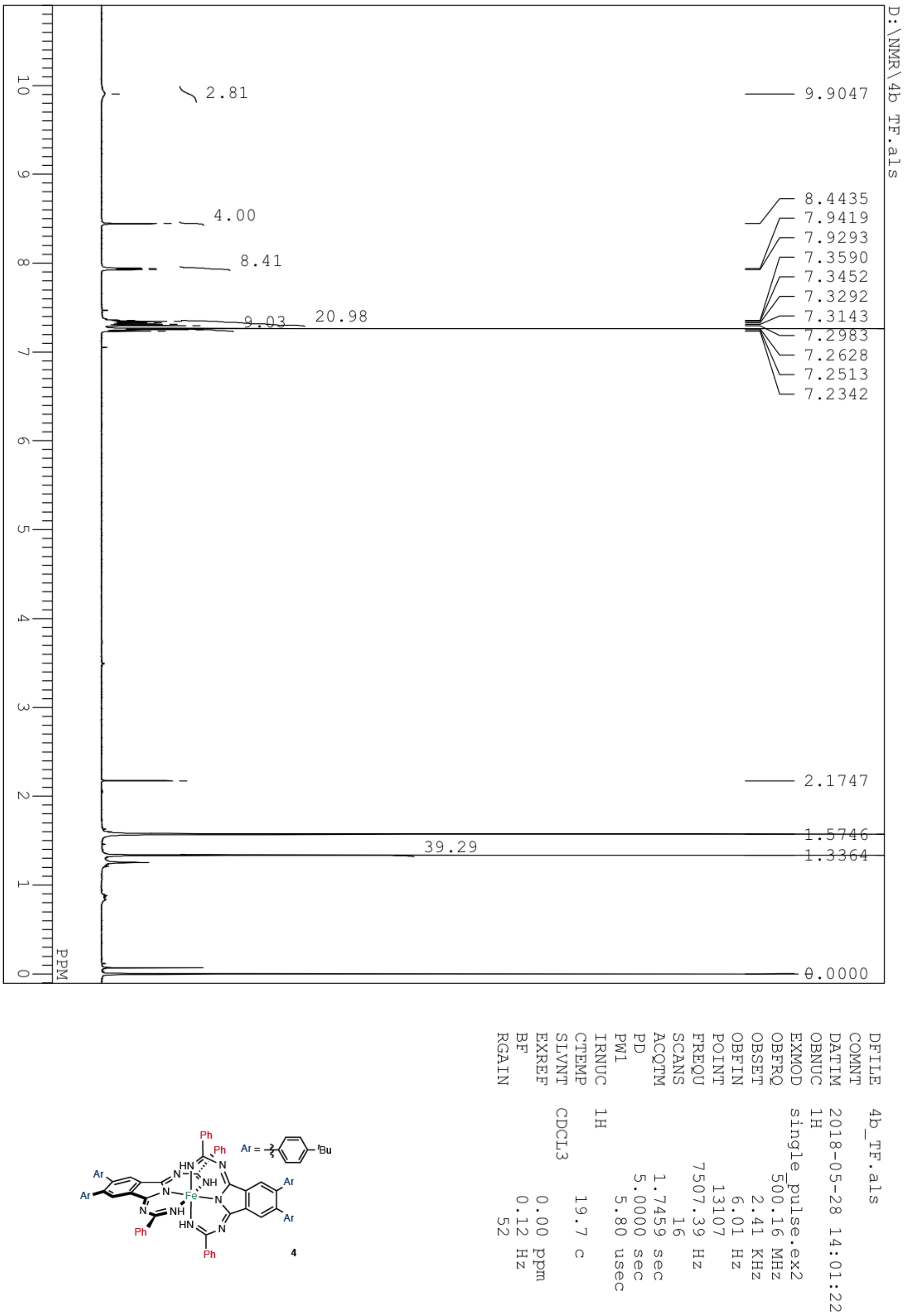


**Supplementary Fig. 18** $^{1}$H NMR spectrum of **4** in $CDCl_3$.

## Full Computational Details

### Computational Details

Geometry optimization for all molecules was performed at the DFT level, by means of the hybrid Becke3LYP[vii] (B3LYP) functional as implemented in Gaussian 2009.[viii] Ru atom was described using a SDD.[ix] The 6-31G(d) basis set was used for the other atoms (denoted as 631SDD). After the geometry optimization, the time-dependent (TD) DFT calculations[x] were performed to evaluate the stick absorption spectrum employing ωB97XD[xi] with the same basis set. Stationary points of **RuTAP'** and, **1a'**, **2a'**, and **1c'** were optimized with $C_2$ and $S_4$ symmetry assumptions. All calculations used a relatively simple self-consistent reaction field (SCRF) method based on the polarizable continuum model (PCM)[xii] that mimicked the solvation effect of chloroform (ε = 4.7113).

### Cartesian Coordinates and Total Electron Energies

**RuTAP'**

SCF Done: E(RB3LYP) = -3492.55757430 A.U.

| Center Number | Atomic Number | Atomic Type | Coordinates (Angstroms) X | Y | Z |
|---|---|---|---|---|---|
| 1 | 44 | 0 | 0.000000 | 0.000000 | 0.029045 |
| 2 | 7 | 0 | 0.000000 | 0.000000 | 2.174436 |
| 3 | 7 | 0 | 3.377106 | 0.026545 | -0.020922 |
| 4 | 7 | 0 | -3.377106 | -0.026545 | -0.020922 |
| 5 | 6 | 0 | 0.000000 | 0.000000 | 4.994082 |
| 6 | 1 | 0 | 0.000000 | 0.000000 | 6.080465 |
| 7 | 7 | 0 | 0.000000 | 0.000000 | -2.116989 |
| 8 | 6 | 0 | 0.000000 | 0.000000 | -4.941801 |
| 9 | 1 | 0 | 0.000000 | 0.000000 | -6.028437 |
| 10 | 7 | 0 | -0.026532 | 3.376346 | 0.049782 |
| 11 | 6 | 0 | 2.756417 | 1.215041 | -0.003102 |
| 12 | 6 | 0 | 3.438419 | 2.523793 | 0.009297 |
| 13 | 6 | 0 | 2.451629 | 3.489329 | 0.040402 |
| 14 | 6 | 0 | 1.163331 | 2.779568 | 0.050481 |
| 15 | 7 | 0 | 1.403918 | 1.431881 | 0.026455 |
| 16 | 6 | 0 | 4.900428 | 2.709024 | 0.060202 |
| 17 | 6 | 0 | 2.596862 | 4.955984 | -0.011812 |
| 18 | 6 | 0 | 5.473959 | 3.608783 | 0.976847 |
| 19 | 6 | 0 | 6.854224 | 3.789165 | 1.032865 |
| 20 | 6 | 0 | 7.691830 | 3.076188 | 0.172631 |
| 21 | 6 | 0 | 7.136071 | 2.178567 | -0.740163 |

| | | | | | |
|---|---|---|---|---|---|
| 22 | 6 | 0 | 5.755286 | 1.992921 | -0.793971 |
| 23 | 6 | 0 | 1.858856 | 5.791381 | 0.844659 |
| 24 | 6 | 0 | 2.004443 | 7.177316 | 0.790489 |
| 25 | 6 | 0 | 2.883875 | 7.758219 | -0.125365 |
| 26 | 6 | 0 | 3.618807 | 6.940026 | -0.987580 |
| 27 | 6 | 0 | 3.477863 | 5.554765 | -0.931052 |
| 28 | 6 | 0 | -0.020556 | 1.163866 | 2.878396 |
| 29 | 6 | 0 | -0.021215 | 1.203102 | 4.275097 |
| 30 | 1 | 0 | 4.828103 | 4.162192 | 1.651378 |
| 31 | 1 | 0 | 7.276483 | 4.485743 | 1.752522 |
| 32 | 1 | 0 | 8.768655 | 3.216824 | 0.216039 |
| 33 | 1 | 0 | 7.779924 | 1.617817 | -1.412738 |
| 34 | 1 | 0 | 5.328779 | 1.292564 | -1.502181 |
| 35 | 1 | 0 | 1.173129 | 5.345591 | 1.556965 |
| 36 | 1 | 0 | 1.428374 | 7.805090 | 1.465585 |
| 37 | 1 | 0 | 2.994163 | 8.838579 | -0.169096 |
| 38 | 1 | 0 | 4.302080 | 7.381542 | -1.708490 |
| 39 | 1 | 0 | 4.049074 | 4.924436 | -1.605474 |
| 40 | 1 | 0 | -0.037135 | 2.076040 | 2.296977 |
| 41 | 1 | 0 | -0.038204 | 2.164225 | 4.779238 |
| 42 | 6 | 0 | -1.206949 | 2.761370 | 0.037853 |
| 43 | 6 | 0 | -2.506376 | 3.450768 | 0.006027 |
| 44 | 6 | 0 | -3.476673 | 2.468772 | -0.026602 |
| 45 | 6 | 0 | -2.774481 | 1.171350 | -0.016590 |
| 46 | 7 | 0 | -1.425960 | 1.409872 | 0.022805 |
| 47 | 6 | 0 | -2.675363 | 4.913936 | -0.066218 |
| 48 | 6 | 0 | -5.767558 | 1.908287 | -0.880244 |
| 49 | 6 | 0 | -1.948087 | 5.773343 | 0.775668 |
| 50 | 6 | 0 | -2.115158 | 7.155859 | 0.701329 |
| 51 | 6 | 0 | -3.006175 | 7.709713 | -0.220014 |
| 52 | 6 | 0 | -3.730553 | 6.867636 | -1.068105 |
| 53 | 6 | 0 | -3.567761 | 5.485710 | -0.991941 |
| 54 | 6 | 0 | -7.151968 | 2.072367 | -0.854499 |
| 55 | 6 | 0 | -7.740550 | 2.952708 | 0.054675 |
| 56 | 6 | 0 | -6.932424 | 3.670732 | 0.938560 |
| 57 | 6 | 0 | -5.548483 | 3.512543 | 0.909811 |
| 58 | 6 | 0 | 0.000000 | 1.206911 | -4.221862 |
| 59 | 6 | 0 | 0.000078 | 1.168386 | -2.822205 |
| 60 | 6 | 0 | -4.942336 | 2.629719 | -0.001882 |
| 61 | 1 | 0 | -5.314731 | 1.223001 | -1.587421 |
| 62 | 1 | 0 | -1.252099 | 5.349114 | 1.490898 |
| 63 | 1 | 0 | -1.545845 | 7.802233 | 1.364457 |
| 64 | 1 | 0 | -3.133155 | 8.787501 | -0.279323 |
| 65 | 1 | 0 | -4.422329 | 7.287813 | -1.793659 |
| 66 | 1 | 0 | -4.130232 | 4.837312 | -1.656390 |
| 67 | 1 | 0 | -7.772782 | 1.509669 | -1.546928 |
| 68 | 1 | 0 | -8.820070 | 3.076624 | 0.076394 |
| 69 | 1 | 0 | -7.380603 | 4.354396 | 1.654899 |
| 70 | 1 | 0 | -4.925011 | 4.070644 | 1.601435 |
| 71 | 1 | 0 | -0.000014 | 2.168593 | -4.725517 |
| 72 | 1 | 0 | 0.000260 | 2.081938 | -2.241809 |
| 73 | 7 | 0 | 0.026532 | -3.376346 | 0.049782 |
| 74 | 6 | 0 | 2.774481 | -1.171350 | -0.016590 |
| 75 | 6 | 0 | 3.476673 | -2.468772 | -0.026602 |
| 76 | 6 | 0 | 2.506376 | -3.450768 | 0.006027 |
| 77 | 6 | 0 | 1.206949 | -2.761370 | 0.037853 |
| 78 | 7 | 0 | 1.425960 | -1.409872 | 0.022805 |
| 79 | 6 | 0 | 4.942336 | -2.629719 | -0.001882 |
| 80 | 6 | 0 | 2.675363 | -4.913936 | -0.066218 |
| 81 | 6 | 0 | 5.548483 | -3.512543 | 0.909811 |
| 82 | 6 | 0 | 6.932424 | -3.670732 | 0.938560 |
| 83 | 6 | 0 | 7.740550 | -2.952708 | 0.054675 |
| 84 | 6 | 0 | 7.151968 | -2.072367 | -0.854499 |
| 85 | 6 | 0 | 5.767558 | -1.908287 | -0.880244 |
| 86 | 6 | 0 | 1.948087 | -5.773343 | 0.775668 |
| 87 | 6 | 0 | 2.115158 | -7.155859 | 0.701329 |
| 88 | 6 | 0 | 3.006175 | -7.709713 | -0.220014 |

| | | | | | |
|---|---|---|---|---|---|
| 89 | 6 | 0 | 3.730553 | -6.867636 | -1.068105 |
| 90 | 6 | 0 | 3.567761 | -5.485710 | -0.991941 |
| 91 | 6 | 0 | 0.020556 | -1.163866 | 2.878396 |
| 92 | 6 | 0 | 0.021215 | -1.203102 | 4.275097 |
| 93 | 1 | 0 | 4.925011 | -4.070644 | 1.601435 |
| 94 | 1 | 0 | 7.380603 | -4.354396 | 1.654899 |
| 95 | 1 | 0 | 8.820070 | -3.076624 | 0.076394 |
| 96 | 1 | 0 | 7.772782 | -1.509669 | -1.546928 |
| 97 | 1 | 0 | 5.314731 | -1.223001 | -1.587421 |
| 98 | 1 | 0 | 1.252099 | -5.349114 | 1.490898 |
| 99 | 1 | 0 | 1.545845 | -7.802233 | 1.364457 |
| 100 | 1 | 0 | 3.133155 | -8.787501 | -0.279323 |
| 101 | 1 | 0 | 4.422329 | -7.287813 | -1.793659 |
| 102 | 1 | 0 | 4.130232 | -4.837312 | -1.656390 |
| 103 | 1 | 0 | 0.037135 | -2.076040 | 2.296977 |
| 104 | 1 | 0 | 0.038204 | -2.164225 | 4.779238 |
| 105 | 6 | 0 | -1.163331 | -2.779568 | 0.050481 |
| 106 | 6 | 0 | -2.451629 | -3.489329 | 0.040402 |
| 107 | 6 | 0 | -3.438419 | -2.523793 | 0.009297 |
| 108 | 6 | 0 | -2.756417 | -1.215041 | -0.003102 |
| 109 | 7 | 0 | -1.403918 | -1.431881 | 0.026455 |
| 110 | 6 | 0 | -2.596862 | -4.955984 | -0.011812 |
| 111 | 6 | 0 | -5.755286 | -1.992921 | -0.793971 |
| 112 | 6 | 0 | -1.858856 | -5.791381 | 0.844659 |
| 113 | 6 | 0 | -2.004443 | -7.177316 | 0.790489 |
| 114 | 6 | 0 | -2.883875 | -7.758219 | -0.125365 |
| 115 | 6 | 0 | -3.618807 | -6.940026 | -0.987580 |
| 116 | 6 | 0 | -3.477863 | -5.554765 | -0.931052 |
| 117 | 6 | 0 | -7.136071 | -2.178567 | -0.740163 |
| 118 | 6 | 0 | -7.691830 | -3.076188 | 0.172631 |
| 119 | 6 | 0 | -6.854224 | -3.789165 | 1.032865 |
| 120 | 6 | 0 | -5.473959 | -3.608783 | 0.976847 |
| 121 | 6 | 0 | -0.000000 | -1.206911 | -4.221862 |
| 122 | 6 | 0 | -0.000078 | -1.168386 | -2.822205 |
| 123 | 6 | 0 | -4.900428 | -2.709024 | 0.060202 |
| 124 | 1 | 0 | -5.328779 | -1.292564 | -1.502181 |
| 125 | 1 | 0 | -1.173129 | -5.345591 | 1.556965 |
| 126 | 1 | 0 | -1.428374 | -7.805090 | 1.465585 |
| 127 | 1 | 0 | -2.994163 | -8.838579 | -0.169096 |
| 128 | 1 | 0 | -4.302080 | -7.381542 | -1.708490 |
| 129 | 1 | 0 | -4.049074 | -4.924436 | -1.605474 |
| 130 | 1 | 0 | -7.779924 | -1.617817 | -1.412738 |
| 131 | 1 | 0 | -8.768655 | -3.216824 | 0.216039 |
| 132 | 1 | 0 | -7.276483 | -4.485743 | 1.752522 |
| 133 | 1 | 0 | -4.828103 | -4.162192 | 1.651378 |
| 134 | 1 | 0 | 0.000014 | -2.168593 | -4.725517 |
| 135 | 1 | 0 | -0.000260 | -2.081938 | -2.241809 |

---------------------------------------------------------------------

**TD–DFT output**

HOMO**: 290**, LUMO**: 291**

Excited State 1: Singlet-A 1.6842 eV 736.18 nm f=0.0000 <S**2>=0.000
283 -> 292 -0.10529
284 -> 291 0.10946
288 -> 292 -0.43760
289 -> 291 0.51811
This state for optimization and/or second-order correction.
Total Energy, E(TD-HF/TD-KS) = -3491.43136322
Copying the excited state density for this state as the 1-particle RhoCI density.

Excited State 2: Singlet-A 1.8271 eV 678.57 nm f=0.0002 <S**2>=0.000
288 -> 291 -0.46855
289 -> 292 0.49260

Excited State 3: Singlet-A 1.9267 eV 643.49 nm f=0.0000 <S**2>=0.000
283 -> 292 0.14076
284 -> 291 0.11608

288 -> 292 0.50470
289 -> 291 0.43377

Excited State 4: Singlet-B 2.1546 eV 575.44 nm f=0.0014 <S**2>=0.000
287 -> 292 0.67963

Excited State 5: Singlet-B 2.1722 eV 570.78 nm f=0.0016 <S**2>=0.000
287 -> 291 0.68278

Excited State 6: Singlet-B 2.4072 eV 515.05 nm f=0.4939 <S**2>=0.000
282 -> 291 0.18522
290 -> 292 0.66837

Excited State 7: Singlet-B 2.4227 eV 511.77 nm f=0.5374 <S**2>=0.000
282 -> 292 -0.18502
290 -> 291 0.66895

Excited State 8: Singlet-A 2.6175 eV 473.68 nm f=0.0000 <S**2>=0.000
288 -> 291 0.49571
289 -> 292 0.48065

Excited State 9: Singlet-B 3.2814 eV 377.84 nm f=0.0984 <S**2>=0.000
282 -> 291 -0.26498
282 -> 292 0.10515
285 -> 291 -0.23857
286 -> 291 -0.21072
286 -> 292 0.38382
288 -> 293 -0.29873
289 -> 293 -0.13984

Excited State 10: Singlet-B 3.2850 eV 377.43 nm f=0.1072 <S**2>=0.000
282 -> 291 -0.10759
282 -> 292 -0.26316
285 -> 291 -0.12171
285 -> 292 -0.25088
286 -> 291 0.38123
286 -> 292 0.18403
288 -> 293 -0.14243
289 -> 293 0.29369

**1a'**

SCF Done: E(RB3LYP) = -2955.45445838 A.U.

| Center Number | Atomic Number | Atomic Type | Coordinates (Angstroms) X | Y | Z |
|---|---|---|---|---|---|
| 1 | 44 | 0 | 0.000000 | 0.000000 | 0.000000 |
| 2 | 7 | 0 | 0.000000 | 2.041107 | 0.096348 |
| 3 | 1 | 0 | 0.032141 | 2.567186 | -0.775081 |
| 4 | 6 | 0 | -0.052752 | 2.809783 | 1.168052 |
| 5 | 6 | 0 | -0.153476 | 4.296941 | 1.024969 |
| 6 | 6 | 0 | -0.613308 | 4.896702 | -0.160184 |
| 7 | 1 | 0 | -0.955081 | 4.285371 | -0.991436 |
| 8 | 6 | 0 | -0.681771 | 6.283752 | -0.279619 |
| 9 | 1 | 0 | -1.047560 | 6.725587 | -1.202516 |
| 10 | 6 | 0 | -0.296471 | 7.099987 | 0.785604 |
| 11 | 1 | 0 | -0.349879 | 8.181356 | 0.692561 |
| 12 | 6 | 0 | 0.143698 | 6.515537 | 1.975133 |
| 13 | 6 | 0 | 0.207757 | 5.128938 | 2.097499 |
| 14 | 1 | 0 | 0.527890 | 4.669251 | 3.024721 |
| 15 | 7 | 0 | -0.017445 | 2.370432 | 2.459067 |
| 16 | 7 | 0 | 0.000000 | 0.000000 | 2.039179 |
| 17 | 6 | 0 | 0.016310 | 1.108926 | 2.829919 |
| 18 | 6 | 0 | 0.020653 | 0.683980 | 4.259172 |
| 19 | 6 | 0 | 0.000304 | 1.621666 | 5.395153 |
| 20 | 6 | 0 | 0.877747 | 2.719808 | 5.438792 |
| 21 | 6 | 0 | 0.860453 | 3.602206 | 6.518511 |

| | | | | | |
|---|---|---|---|---|---|
| 22 | 6 | 0 | -0.039514 | 3.411822 | 7.569214 |
| 23 | 6 | 0 | -0.920849 | 2.328540 | 7.534267 |
| 24 | 6 | 0 | -0.899968 | 1.440892 | 6.460116 |
| 25 | 1 | 0 | 1.552246 | 4.440399 | 6.538835 |
| 26 | 1 | 0 | -1.629007 | 2.175263 | 8.344356 |
| 27 | 1 | 0 | -1.589759 | 0.603059 | 6.435977 |
| 28 | 1 | 0 | 1.577045 | 2.871627 | 4.623684 |
| 29 | 1 | 0 | 0.434893 | 7.142022 | 2.814124 |
| 30 | 7 | 0 | 0.000000 | -2.041107 | 0.096348 |
| 31 | 1 | 0 | -0.032141 | -2.567186 | -0.775081 |
| 32 | 6 | 0 | 0.052752 | -2.809783 | 1.168052 |
| 33 | 6 | 0 | 0.153476 | -4.296941 | 1.024969 |
| 34 | 6 | 0 | 0.613308 | -4.896702 | -0.160184 |
| 35 | 1 | 0 | 0.955081 | -4.285371 | -0.991436 |
| 36 | 6 | 0 | 0.681771 | -6.283752 | -0.279619 |
| 37 | 1 | 0 | 1.047560 | -6.725587 | -1.202516 |
| 38 | 6 | 0 | 0.296471 | -7.099987 | 0.785604 |
| 39 | 1 | 0 | 0.349879 | -8.181356 | 0.692561 |
| 40 | 6 | 0 | -0.143698 | -6.515537 | 1.975133 |
| 41 | 6 | 0 | -0.207757 | -5.128938 | 2.097499 |
| 42 | 1 | 0 | -0.527890 | -4.669251 | 3.024721 |
| 43 | 7 | 0 | 0.017445 | -2.370432 | 2.459067 |
| 44 | 6 | 0 | -0.016310 | -1.108926 | 2.829919 |
| 45 | 6 | 0 | -0.020653 | -0.683980 | 4.259172 |
| 46 | 6 | 0 | -0.000304 | -1.621666 | 5.395153 |
| 47 | 6 | 0 | -0.877747 | -2.719808 | 5.438792 |
| 48 | 6 | 0 | -0.860453 | -3.602206 | 6.518511 |
| 49 | 6 | 0 | 0.039514 | -3.411822 | 7.569214 |
| 50 | 6 | 0 | 0.920849 | -2.328540 | 7.534267 |
| 51 | 6 | 0 | 0.899968 | -1.440892 | 6.460116 |
| 52 | 1 | 0 | -1.552246 | -4.440399 | 6.538835 |
| 53 | 1 | 0 | 1.629007 | -2.175263 | 8.344356 |
| 54 | 1 | 0 | 1.589759 | -0.603059 | 6.435977 |
| 55 | 1 | 0 | -1.577045 | -2.871627 | 4.623684 |
| 56 | 1 | 0 | -0.434893 | -7.142022 | 2.814124 |
| 57 | 7 | 0 | -2.041107 | 0.000000 | -0.096348 |
| 58 | 1 | 0 | -2.567186 | 0.032141 | 0.775081 |
| 59 | 6 | 0 | -2.809783 | -0.052752 | -1.168052 |
| 60 | 6 | 0 | -4.296941 | -0.153476 | -1.024969 |
| 61 | 6 | 0 | -4.896702 | -0.613308 | 0.160184 |
| 62 | 1 | 0 | -4.285371 | -0.955081 | 0.991436 |
| 63 | 6 | 0 | -6.283752 | -0.681771 | 0.279619 |
| 64 | 1 | 0 | -6.725587 | -1.047560 | 1.202516 |
| 65 | 6 | 0 | -7.099987 | -0.296471 | -0.785604 |
| 66 | 1 | 0 | -8.181356 | -0.349879 | -0.692561 |
| 67 | 6 | 0 | -6.515537 | 0.143698 | -1.975133 |
| 68 | 6 | 0 | -5.128938 | 0.207757 | -2.097499 |
| 69 | 1 | 0 | -4.669251 | 0.527890 | -3.024721 |
| 70 | 7 | 0 | -2.370432 | -0.017445 | -2.459067 |
| 71 | 7 | 0 | 0.000000 | 0.000000 | -2.039179 |
| 72 | 6 | 0 | -1.108926 | 0.016310 | -2.829919 |
| 73 | 6 | 0 | -0.683980 | 0.020653 | -4.259172 |
| 74 | 6 | 0 | -1.621666 | 0.000304 | -5.395153 |
| 75 | 6 | 0 | -2.719808 | 0.877747 | -5.438792 |
| 76 | 6 | 0 | -3.602206 | 0.860453 | -6.518511 |
| 77 | 6 | 0 | -3.411822 | -0.039514 | -7.569214 |
| 78 | 6 | 0 | -2.328540 | -0.920849 | -7.534267 |
| 79 | 6 | 0 | -1.440892 | -0.899968 | -6.460116 |
| 80 | 1 | 0 | -4.440399 | 1.552246 | -6.538835 |
| 81 | 1 | 0 | -2.175263 | -1.629007 | -8.344356 |
| 82 | 1 | 0 | -0.603059 | -1.589759 | -6.435977 |
| 83 | 1 | 0 | -2.871627 | 1.577045 | -4.623684 |
| 84 | 1 | 0 | -7.142022 | 0.434893 | -2.814124 |
| 85 | 7 | 0 | 2.041107 | 0.000000 | -0.096348 |
| 86 | 1 | 0 | 2.567186 | -0.032141 | 0.775081 |
| 87 | 6 | 0 | 2.809783 | 0.052752 | -1.168052 |
| 88 | 6 | 0 | 4.296941 | 0.153476 | -1.024969 |

| | | | | | |
|---|---|---|---|---|---|
| 89 | 6 | 0 | 4.896702 | 0.613308 | 0.160184 |
| 90 | 1 | 0 | 4.285371 | 0.955081 | 0.991436 |
| 91 | 6 | 0 | 6.283752 | 0.681771 | 0.279619 |
| 92 | 1 | 0 | 6.725587 | 1.047560 | 1.202516 |
| 93 | 6 | 0 | 7.099987 | 0.296471 | -0.785604 |
| 94 | 1 | 0 | 8.181356 | 0.349879 | -0.692561 |
| 95 | 6 | 0 | 6.515537 | -0.143698 | -1.975133 |
| 96 | 6 | 0 | 5.128938 | -0.207757 | -2.097499 |
| 97 | 1 | 0 | 4.669251 | -0.527890 | -3.024721 |
| 98 | 7 | 0 | 2.370432 | 0.017445 | -2.459067 |
| 99 | 6 | 0 | 1.108926 | -0.016310 | -2.829919 |
| 100 | 6 | 0 | 0.683980 | -0.020653 | -4.259172 |
| 101 | 6 | 0 | 1.621666 | -0.000304 | -5.395153 |
| 102 | 6 | 0 | 2.719808 | -0.877747 | -5.438792 |
| 103 | 6 | 0 | 3.602206 | -0.860453 | -6.518511 |
| 104 | 6 | 0 | 3.411822 | 0.039514 | -7.569214 |
| 105 | 6 | 0 | 2.328540 | 0.920849 | -7.534267 |
| 106 | 6 | 0 | 1.440892 | 0.899968 | -6.460116 |
| 107 | 1 | 0 | 4.440399 | -1.552246 | -6.538835 |
| 108 | 1 | 0 | 2.175263 | 1.629007 | -8.344356 |
| 109 | 1 | 0 | 0.603059 | 1.589759 | -6.435977 |
| 110 | 1 | 0 | 2.871627 | -1.577045 | -4.623684 |
| 111 | 1 | 0 | 7.142022 | -0.434893 | -2.814124 |
| 112 | 1 | 0 | 0.055285 | -4.102666 | 8.408004 |
| 113 | 1 | 0 | -0.055285 | 4.102666 | 8.408004 |
| 114 | 1 | 0 | -4.102666 | -0.055285 | -8.408004 |
| 115 | 1 | 0 | 4.102666 | 0.055285 | -8.408004 |

---------------------------------------------------------------------

**TD–DFT output**

HOMO**: 245**, LUMO**: 246**

Excited State 1: Singlet-E 1.7824 eV 695.59 nm f=0.0091 <S**2>=0.000
243 -> 246 0.14591
243 -> 247 0.19271
244 -> 246 0.59948
244 -> 247 0.24555
This state for optimization and/or second-order correction.
Total Energy, E(TD-HF/TD-KS) = -2954.47138924
Copying the excited state density for this state as the 1-particle RhoCI density.

Excited State 2: Singlet-E 1.7824 eV 695.59 nm f=0.0091 <S**2>=0.000
243 -> 246 0.59948
243 -> 247 -0.24555
244 -> 246 -0.14591
244 -> 247 0.19271

Excited State 3: Singlet-E 1.9029 eV 651.56 nm f=0.0004 <S**2>=0.000
243 -> 246 -0.19724
243 -> 247 0.10938
244 -> 246 -0.22908
244 -> 247 0.57515
244 -> 251 0.12603

Excited State 4: Singlet-E 1.9029 eV 651.56 nm f=0.0004 <S**2>=0.000
243 -> 246 0.22908
243 -> 247 0.57515
243 -> 251 0.12603
244 -> 246 -0.19724
244 -> 247 -0.10938

Excited State 5: Singlet-B 1.9593 eV 632.80 nm f=0.3477 <S**2>=0.000
245 -> 246 0.69484

Excited State 6: Singlet-A 2.7362 eV 453.13 nm f=0.0000 <S**2>=0.000
245 -> 247 0.68462

Excited State 7: Singlet-E 3.3983 eV 364.84 nm f=0.1354 <S**2>=0.000

220 -> 246 -0.10687
239 -> 246 -0.13397
241 -> 246 0.52345
241 -> 247 0.33293
245 -> 248 -0.11622

Excited State 8: Singlet-E 3.3983 eV 364.84 nm f=0.1354 <S**2>=0.000
219 -> 246 -0.10687
240 -> 246 0.13397
242 -> 246 0.52345
242 -> 247 -0.33294
245 -> 249 0.11623

Excited State 9: Singlet-E 3.6205 eV 342.45 nm f=0.0159 <S**2>=0.000
224 -> 246 0.10083
224 -> 247 -0.16958
234 -> 246 -0.14175
239 -> 246 0.45308
239 -> 247 0.12126
240 -> 246 -0.22071
241 -> 246 0.13576
245 -> 248 -0.28776

Excited State 10: Singlet-E 3.6205 eV 342.45 nm f=0.0159 <S**2>=0.000
223 -> 246 -0.10079
223 -> 247 -0.16955
235 -> 246 0.14171
239 -> 246 0.22072
240 -> 246 0.45307
240 -> 247 -0.12126
242 -> 246 -0.13574
245 -> 249 -0.28780

**2a'**

SCF Done: E(RB3LYP) = -3262.74436308 A.U.

| Center Number | Atomic Number | Atomic Type | Coordinates (Angstroms) X | Y | Z |
|---|---|---|---|---|---|
| 1 | 44 | 0 | 0.000000 | 0.000000 | 0.000000 |
| 2 | 7 | 0 | 0.000000 | 2.046291 | 0.085908 |
| 3 | 7 | 0 | 0.000000 | 0.000000 | 2.033067 |
| 4 | 7 | 0 | -0.000000 | -2.046291 | 0.085908 |
| 5 | 6 | 0 | 0.000000 | 2.826399 | 1.147228 |
| 6 | 7 | 0 | 0.000000 | 2.384233 | 2.440310 |
| 7 | 6 | 0 | 0.000000 | 1.125884 | 2.813206 |
| 8 | 6 | 0 | 0.000000 | -5.122267 | 2.163811 |
| 9 | 6 | 0 | 0.000000 | 0.701077 | 4.229993 |
| 10 | 6 | 0 | -0.000000 | -0.701077 | 4.229993 |
| 11 | 6 | 0 | -0.000000 | -1.125884 | 2.813206 |
| 12 | 7 | 0 | -0.000000 | -2.384233 | 2.440310 |
| 13 | 6 | 0 | -0.000000 | -2.826399 | 1.147228 |
| 14 | 6 | 0 | 0.000000 | 4.320993 | 1.009158 |
| 15 | 6 | 0 | 0.000000 | 4.965687 | -0.240775 |
| 16 | 6 | 0 | 0.000000 | 6.355974 | -0.331981 |
| 17 | 6 | 0 | 0.000000 | 7.137931 | 0.824853 |
| 18 | 6 | 0 | 0.000000 | 6.512641 | 2.072627 |
| 19 | 6 | 0 | 0.000000 | 5.122267 | 2.163811 |
| 20 | 6 | 0 | -0.000000 | -4.320993 | 1.009158 |
| 21 | 6 | 0 | -0.000000 | -6.512641 | 2.072627 |
| 22 | 6 | 0 | -0.000000 | -7.137931 | 0.824853 |
| 23 | 6 | 0 | -0.000000 | -6.355974 | -0.331981 |
| 24 | 6 | 0 | -0.000000 | -4.965687 | -0.240775 |
| 25 | 7 | 0 | 2.046291 | -0.000000 | -0.085908 |
| 26 | 7 | 0 | 0.000000 | 0.000000 | -2.033067 |
| 27 | 7 | 0 | -2.046291 | 0.000000 | -0.085908 |
| 28 | 6 | 0 | 2.826399 | -0.000000 | -1.147228 |

| | | | | | |
|---|---|---|---|---|---|
| 29 | 7 | 0 | 2.384233 | -0.000000 | -2.440310 |
| 30 | 6 | 0 | 1.125884 | -0.000000 | -2.813206 |
| 31 | 6 | 0 | -5.122267 | 0.000000 | -2.163811 |
| 32 | 6 | 0 | 0.701077 | 0.000000 | -4.229993 |
| 33 | 6 | 0 | -0.701077 | 0.000000 | -4.229993 |
| 34 | 6 | 0 | -1.125884 | 0.000000 | -2.813206 |
| 35 | 7 | 0 | -2.384233 | 0.000000 | -2.440310 |
| 36 | 6 | 0 | -2.826399 | 0.000000 | -1.147228 |
| 37 | 6 | 0 | 4.320993 | -0.000000 | -1.009158 |
| 38 | 6 | 0 | 4.965687 | -0.000000 | 0.240775 |
| 39 | 6 | 0 | 6.355974 | -0.000000 | 0.331981 |
| 40 | 6 | 0 | 7.137931 | -0.000000 | -0.824853 |
| 41 | 6 | 0 | 6.512641 | -0.000000 | -2.072627 |
| 42 | 6 | 0 | 5.122267 | -0.000000 | -2.163811 |
| 43 | 6 | 0 | -4.320993 | 0.000000 | -1.009158 |
| 44 | 6 | 0 | -6.512641 | 0.000000 | -2.072627 |
| 45 | 6 | 0 | -7.137931 | 0.000000 | -0.824853 |
| 46 | 6 | 0 | -6.355974 | 0.000000 | 0.331981 |
| 47 | 6 | 0 | -4.965687 | 0.000000 | 0.240775 |
| 48 | 6 | 0 | 0.000000 | 1.412938 | 5.420399 |
| 49 | 6 | 0 | 0.000000 | 0.708857 | 6.638653 |
| 50 | 6 | 0 | -0.000000 | -0.708857 | 6.638653 |
| 51 | 6 | 0 | -0.000000 | -1.412938 | 5.420399 |
| 52 | 6 | 0 | 1.412938 | -0.000000 | -5.420399 |
| 53 | 6 | 0 | 0.708857 | 0.000000 | -6.638653 |
| 54 | 6 | 0 | -0.708857 | 0.000000 | -6.638653 |
| 55 | 6 | 0 | -1.412938 | 0.000000 | -5.420399 |
| 56 | 1 | 0 | 0.000000 | -4.630940 | 3.128465 |
| 57 | 1 | 0 | 0.000000 | 4.397501 | -1.167156 |
| 58 | 1 | 0 | 0.000000 | 6.827753 | -1.310828 |
| 59 | 1 | 0 | 0.000000 | 8.222181 | 0.752449 |
| 60 | 1 | 0 | 0.000000 | 7.108787 | 2.981382 |
| 61 | 1 | 0 | 0.000000 | 4.630940 | 3.128465 |
| 62 | 1 | 0 | -0.000000 | -7.108787 | 2.981382 |
| 63 | 1 | 0 | -0.000000 | -8.222181 | 0.752449 |
| 64 | 1 | 0 | -0.000000 | -6.827753 | -1.310828 |
| 65 | 1 | 0 | -0.000000 | -4.397501 | -1.167156 |
| 66 | 1 | 0 | -4.630940 | 0.000000 | -3.128465 |
| 67 | 1 | 0 | 4.397501 | -0.000000 | 1.167156 |
| 68 | 1 | 0 | 6.827753 | -0.000000 | 1.310828 |
| 69 | 1 | 0 | 8.222181 | -0.000000 | -0.752449 |
| 70 | 1 | 0 | 7.108787 | -0.000000 | -2.981382 |
| 71 | 1 | 0 | 4.630940 | -0.000000 | -3.128465 |
| 72 | 1 | 0 | -7.108787 | 0.000000 | -2.981382 |
| 73 | 1 | 0 | -8.222181 | 0.000000 | -0.752449 |
| 74 | 1 | 0 | -6.827753 | 0.000000 | 1.310828 |
| 75 | 1 | 0 | -4.397501 | 0.000000 | 1.167156 |
| 76 | 1 | 0 | 0.000000 | 2.498538 | 5.418539 |
| 77 | 1 | 0 | -0.000000 | -2.498538 | 5.418539 |
| 78 | 1 | 0 | 2.498538 | -0.000000 | -5.418539 |
| 79 | 1 | 0 | -2.498538 | 0.000000 | -5.418539 |
| 80 | 6 | 0 | -1.492329 | 0.000000 | -7.915656 |
| 81 | 6 | 0 | -1.882892 | 1.206128 | -8.514513 |
| 82 | 6 | 0 | -1.882892 | -1.206128 | -8.514513 |
| 83 | 6 | 0 | -2.639210 | 1.206776 | -9.687686 |
| 84 | 1 | 0 | -1.592090 | 2.147249 | -8.055395 |
| 85 | 6 | 0 | -2.639210 | -1.206776 | -9.687686 |
| 86 | 1 | 0 | -1.592090 | -2.147249 | -8.055395 |
| 87 | 6 | 0 | -3.018977 | 0.000000 | -10.278030 |
| 88 | 1 | 0 | -2.932810 | 2.150961 | -10.139083 |
| 89 | 1 | 0 | -2.932810 | -2.150961 | -10.139083 |
| 90 | 1 | 0 | -3.608200 | 0.000000 | -11.191212 |
| 91 | 6 | 0 | 1.492329 | 0.000000 | -7.915656 |
| 92 | 6 | 0 | 1.882892 | -1.206128 | -8.514513 |
| 93 | 6 | 0 | 1.882892 | 1.206128 | -8.514513 |
| 94 | 6 | 0 | 2.639210 | -1.206776 | -9.687686 |
| 95 | 1 | 0 | 1.592090 | -2.147249 | -8.055395 |

| | | | | | |
|---|---|---|---|---|---|
| 96 | 6 | 0 | 2.639210 | 1.206776 | -9.687686 |
| 97 | 1 | 0 | 1.592090 | 2.147249 | -8.055395 |
| 98 | 6 | 0 | 3.018977 | 0.000000 | -10.278030 |
| 99 | 1 | 0 | 2.932810 | -2.150961 | -10.139083 |
| 100 | 1 | 0 | 2.932810 | 2.150961 | -10.139083 |
| 101 | 1 | 0 | 3.608200 | 0.000000 | -11.191212 |
| 102 | 6 | 0 | 0.000000 | -1.492329 | 7.915656 |
| 103 | 6 | 0 | 1.206128 | -1.882892 | 8.514513 |
| 104 | 6 | 0 | -1.206128 | -1.882892 | 8.514513 |
| 105 | 6 | 0 | 1.206776 | -2.639210 | 9.687686 |
| 106 | 1 | 0 | 2.147249 | -1.592090 | 8.055395 |
| 107 | 6 | 0 | -1.206776 | -2.639210 | 9.687686 |
| 108 | 1 | 0 | -2.147249 | -1.592090 | 8.055395 |
| 109 | 6 | 0 | 0.000000 | -3.018977 | 10.278030 |
| 110 | 1 | 0 | 2.150961 | -2.932810 | 10.139083 |
| 111 | 1 | 0 | -2.150961 | -2.932810 | 10.139083 |
| 112 | 1 | 0 | -0.000000 | -3.608200 | 11.191212 |
| 113 | 6 | 0 | -0.000000 | 1.492329 | 7.915656 |
| 114 | 6 | 0 | -1.206128 | 1.882892 | 8.514513 |
| 115 | 6 | 0 | 1.206128 | 1.882892 | 8.514513 |
| 116 | 6 | 0 | -1.206776 | 2.639210 | 9.687686 |
| 117 | 1 | 0 | -2.147249 | 1.592090 | 8.055395 |
| 118 | 6 | 0 | 1.206776 | 2.639210 | 9.687686 |
| 119 | 1 | 0 | 2.147249 | 1.592090 | 8.055395 |
| 120 | 6 | 0 | -0.000000 | 3.018977 | 10.278030 |
| 121 | 1 | 0 | -2.150961 | 2.932810 | 10.139083 |
| 122 | 1 | 0 | 2.150961 | 2.932810 | 10.139083 |
| 123 | 1 | 0 | -0.000000 | 3.608200 | 11.191212 |
| 124 | 1 | 0 | 2.552587 | -0.000000 | 0.796068 |
| 125 | 1 | 0 | -2.552587 | -0.000000 | 0.796068 |
| 126 | 1 | 0 | 0.000000 | 2.552587 | -0.796068 |
| 127 | 1 | 0 | -0.000000 | -2.552587 | -0.796068 |

---------------------------------------------------------------------

**TD–DFT output**

HOMO**: 271**, LUMO**: 272**

```
Excited State   1:      Singlet-E      2.0802 eV  596.03 nm  f=0.0303  <S**2>=0.000
     269 -> 272         0.59973
     269 -> 273         0.34620
This state for optimization and/or second-order correction.
Total Energy, E(TD-HF/TD-KS) =  -3261.64760389
Copying the excited state density for this state as the 1-particle RhoCI density.

Excited State   2:      Singlet-E      2.0802 eV  596.03 nm  f=0.0303  <S**2>=0.000
     270 -> 272         0.59974
     270 -> 273        -0.34619

Excited State   3:      Singlet-B2     2.1134 eV  586.65 nm  f=0.4167  <S**2>=0.000
     271 -> 272         0.69533

Excited State   4:      Singlet-E      2.1272 eV  582.86 nm  f=0.0007  <S**2>=0.000
     270 -> 272         0.34446
     270 -> 273         0.57967

Excited State   5:      Singlet-E      2.1272 eV  582.86 nm  f=0.0007  <S**2>=0.000
     269 -> 272        -0.34447
     269 -> 273         0.57966

Excited State   6:      Singlet-A1     2.8249 eV  438.90 nm  f=0.0000  <S**2>=0.000
     271 -> 273         0.67772

Excited State   7:      Singlet-E      3.6019 eV  344.22 nm  f=0.1819  <S**2>=0.000
     267 -> 272        -0.22326
     267 -> 273         0.14532
     271 -> 274         0.61973
     271 -> 298        -0.12811
```

| Excited State | 8: | Singlet-E | 3.6019 eV | 344.22 nm | f=0.1819 | <S**2>=0.000 |
|---|---|---|---|---|---|---|
| | 268 -> 272 | 0.22326 | | | | |
| | 268 -> 273 | 0.14532 | | | | |
| | 271 -> 275 | 0.61973 | | | | |
| | 271 -> 299 | -0.12811 | | | | |

| Excited State | 9: | Singlet-E | 3.7474 eV | 330.85 nm | f=0.0000 | <S**2>=0.000 |
|---|---|---|---|---|---|---|
| | 269 -> 274 | 0.46841 | | | | |
| | 269 -> 298 | -0.12476 | | | | |
| | 270 -> 275 | 0.46840 | | | | |
| | 270 -> 299 | -0.12475 | | | | |

| Excited State | 10: | Singlet-E | 3.7644 eV | 329.36 nm | f=0.0000 | <S**2>=0.000 |
|---|---|---|---|---|---|---|
| | 269 -> 274 | -0.46749 | | | | |
| | 269 -> 298 | 0.12378 | | | | |
| | 270 -> 275 | 0.46751 | | | | |
| | 270 -> 299 | -0.12378 | | | | |

**1c'**

SCF Done:  E(RB3LYP) =  -3019.59562744     A.U.

| Center Number | Atomic Number | Atomic Type | Coordinates (Angstroms) X | Y | Z |
|---|---|---|---|---|---|
| 1 | 44 | 0 | 0.000000 | 0.000000 | 0.000000 |
| 2 | 7 | 0 | 0.000000 | -2.040276 | 0.094599 |
| 3 | 1 | 0 | -0.022396 | -2.565094 | -0.777916 |
| 4 | 6 | 0 | 0.041533 | -2.805200 | 1.168488 |
| 5 | 6 | 0 | 0.125241 | -4.295820 | 1.030417 |
| 6 | 6 | 0 | 0.469966 | -4.930046 | -0.172796 |
| 7 | 1 | 0 | 0.731186 | -4.369577 | -1.066093 |
| 8 | 6 | 0 | 0.513781 | -6.322613 | -0.222079 |
| 9 | 1 | 0 | 0.783261 | -6.823988 | -1.150226 |
| 10 | 6 | 0 | -0.066501 | -6.507201 | 1.971952 |
| 11 | 6 | 0 | -0.135320 | -5.124158 | 2.130919 |
| 12 | 1 | 0 | -0.376466 | -4.683779 | 3.090279 |
| 13 | 7 | 0 | 0.009647 | -2.368589 | 2.458113 |
| 14 | 7 | 0 | 0.000000 | 0.000000 | 2.039479 |
| 15 | 6 | 0 | -0.018217 | -1.107810 | 2.831897 |
| 16 | 6 | 0 | -0.021553 | -0.684026 | 4.261302 |
| 17 | 6 | 0 | -0.000729 | -1.623416 | 5.395605 |
| 18 | 6 | 0 | -0.878301 | -2.721703 | 5.438179 |
| 19 | 6 | 0 | -0.858317 | -3.606457 | 6.515907 |
| 20 | 6 | 0 | 0.044487 | -3.417876 | 7.564584 |
| 21 | 6 | 0 | 0.925269 | -2.334148 | 7.530668 |
| 22 | 6 | 0 | 0.901548 | -1.443650 | 6.458977 |
| 23 | 1 | 0 | -1.550352 | -4.444241 | 6.536771 |
| 24 | 1 | 0 | 1.635113 | -2.182646 | 8.339398 |
| 25 | 1 | 0 | 1.590882 | -0.605428 | 6.435747 |
| 26 | 1 | 0 | -1.580803 | -2.871836 | 4.625382 |
| 27 | 1 | 0 | -0.273165 | -7.161481 | 2.817315 |
| 28 | 7 | 0 | 0.000000 | 2.040276 | 0.094599 |
| 29 | 1 | 0 | 0.022396 | 2.565094 | -0.777916 |
| 30 | 6 | 0 | -0.041533 | 2.805200 | 1.168488 |
| 31 | 6 | 0 | -0.125241 | 4.295820 | 1.030417 |
| 32 | 6 | 0 | -0.469966 | 4.930046 | -0.172796 |
| 33 | 1 | 0 | -0.731186 | 4.369577 | -1.066093 |
| 34 | 6 | 0 | -0.513781 | 6.322613 | -0.222079 |
| 35 | 1 | 0 | -0.783261 | 6.823988 | -1.150226 |
| 36 | 6 | 0 | 0.066501 | 6.507201 | 1.971952 |
| 37 | 6 | 0 | 0.135320 | 5.124158 | 2.130919 |
| 38 | 1 | 0 | 0.376466 | 4.683779 | 3.090279 |
| 39 | 7 | 0 | -0.009647 | 2.368589 | 2.458113 |
| 40 | 6 | 0 | 0.018217 | 1.107810 | 2.831897 |
| 41 | 6 | 0 | 0.021553 | 0.684026 | 4.261302 |
| 42 | 6 | 0 | 0.000729 | 1.623416 | 5.395605 |
| 43 | 6 | 0 | 0.878301 | 2.721703 | 5.438179 |

| | | | | | |
|---|---|---|---|---|---|
| 44 | 6 | 0 | 0.858317 | 3.606457 | 6.515907 |
| 45 | 6 | 0 | -0.044487 | 3.417876 | 7.564584 |
| 46 | 6 | 0 | -0.925269 | 2.334148 | 7.530668 |
| 47 | 6 | 0 | -0.901548 | 1.443650 | 6.458977 |
| 48 | 1 | 0 | 1.550352 | 4.444241 | 6.536771 |
| 49 | 1 | 0 | -1.635113 | 2.182646 | 8.339398 |
| 50 | 1 | 0 | -1.590882 | 0.605428 | 6.435747 |
| 51 | 1 | 0 | 1.580803 | 2.871836 | 4.625382 |
| 52 | 1 | 0 | 0.273165 | 7.161481 | 2.817315 |
| 53 | 7 | 0 | 2.040276 | 0.000000 | -0.094599 |
| 54 | 1 | 0 | 2.565094 | -0.022396 | 0.777916 |
| 55 | 6 | 0 | 2.805200 | 0.041533 | -1.168488 |
| 56 | 6 | 0 | 4.295820 | 0.125241 | -1.030417 |
| 57 | 6 | 0 | 4.930046 | 0.469966 | 0.172796 |
| 58 | 1 | 0 | 4.369577 | 0.731186 | 1.066093 |
| 59 | 6 | 0 | 6.322613 | 0.513781 | 0.222079 |
| 60 | 1 | 0 | 6.823988 | 0.783261 | 1.150226 |
| 61 | 6 | 0 | 6.507201 | -0.066501 | -1.971952 |
| 62 | 6 | 0 | 5.124158 | -0.135320 | -2.130919 |
| 63 | 1 | 0 | 4.683779 | -0.376466 | -3.090279 |
| 64 | 7 | 0 | 2.368589 | 0.009647 | -2.458113 |
| 65 | 7 | 0 | 0.000000 | 0.000000 | -2.039479 |
| 66 | 6 | 0 | 1.107810 | -0.018217 | -2.831897 |
| 67 | 6 | 0 | 0.684026 | -0.021553 | -4.261302 |
| 68 | 6 | 0 | 1.623416 | -0.000729 | -5.395605 |
| 69 | 6 | 0 | 2.721703 | -0.878301 | -5.438179 |
| 70 | 6 | 0 | 3.606457 | -0.858317 | -6.515907 |
| 71 | 6 | 0 | 3.417876 | 0.044487 | -7.564584 |
| 72 | 6 | 0 | 2.334148 | 0.925269 | -7.530668 |
| 73 | 6 | 0 | 1.443650 | 0.901548 | -6.458977 |
| 74 | 1 | 0 | 4.444241 | -1.550352 | -6.536771 |
| 75 | 1 | 0 | 2.182646 | 1.635113 | -8.339398 |
| 76 | 1 | 0 | 0.605428 | 1.590882 | -6.435747 |
| 77 | 1 | 0 | 2.871836 | -1.580803 | -4.625382 |
| 78 | 1 | 0 | 7.161481 | -0.273165 | -2.817315 |
| 79 | 7 | 0 | -2.040276 | 0.000000 | -0.094599 |
| 80 | 1 | 0 | -2.565094 | 0.022396 | 0.777916 |
| 81 | 6 | 0 | -2.805200 | -0.041533 | -1.168488 |
| 82 | 6 | 0 | -4.295820 | -0.125241 | -1.030417 |
| 83 | 6 | 0 | -4.930046 | -0.469966 | 0.172796 |
| 84 | 1 | 0 | -4.369577 | -0.731186 | 1.066093 |
| 85 | 6 | 0 | -6.322613 | -0.513781 | 0.222079 |
| 86 | 1 | 0 | -6.823988 | -0.783261 | 1.150226 |
| 87 | 6 | 0 | -6.507201 | 0.066501 | -1.971952 |
| 88 | 6 | 0 | -5.124158 | 0.135320 | -2.130919 |
| 89 | 1 | 0 | -4.683779 | 0.376466 | -3.090279 |
| 90 | 7 | 0 | -2.368589 | -0.009647 | -2.458113 |
| 91 | 6 | 0 | -1.107810 | 0.018217 | -2.831897 |
| 92 | 6 | 0 | -0.684026 | 0.021553 | -4.261302 |
| 93 | 6 | 0 | -1.623416 | 0.000729 | -5.395605 |
| 94 | 6 | 0 | -2.721703 | 0.878301 | -5.438179 |
| 95 | 6 | 0 | -3.606457 | 0.858317 | -6.515907 |
| 96 | 6 | 0 | -3.417876 | -0.044487 | -7.564584 |
| 97 | 6 | 0 | -2.334148 | -0.925269 | -7.530668 |
| 98 | 6 | 0 | -1.443650 | -0.901548 | -6.458977 |
| 99 | 1 | 0 | -4.444241 | 1.550352 | -6.536771 |
| 100 | 1 | 0 | -2.182646 | -1.635113 | -8.339398 |
| 101 | 1 | 0 | -0.605428 | -1.590882 | -6.435747 |
| 102 | 1 | 0 | -2.871836 | 1.580803 | -4.625382 |
| 103 | 1 | 0 | -7.161481 | 0.273165 | -2.817315 |
| 104 | 1 | 0 | -0.062474 | 4.110711 | 8.401504 |
| 105 | 1 | 0 | 0.062474 | -4.110711 | 8.401504 |
| 106 | 1 | 0 | 4.110711 | 0.062474 | -8.401504 |
| 107 | 1 | 0 | -4.110711 | -0.062474 | -8.401504 |
| 108 | 7 | 0 | 0.247475 | -7.116915 | 0.821477 |
| 109 | 7 | 0 | -0.247475 | 7.116915 | 0.821477 |
| 110 | 7 | 0 | -7.116915 | -0.247475 | -0.821477 |

```
   111          7           0        7.116915    0.247475   -0.821477
 ---------------------------------------------------------------------
```

**TD–DFT output**

HOMO**: 245**, LUMO**: 246**

```
 Excited State   1:      Singlet-E      1.7941 eV  691.07 nm  f=0.0071  <S**2>=0.000
     243 -> 246         0.47364
     243 -> 247         0.24033
     244 -> 246         0.40546
     244 -> 247         0.17431
 This state for optimization and/or second-order correction.
 Total Energy, E(TD-HF/TD-KS) =  -3018.59523516
 Copying the excited state density for this state as the 1-particle RhoCI density.

 Excited State   2:      Singlet-E      1.7941 eV  691.07 nm  f=0.0071  <S**2>=0.000
     243 -> 246        -0.40546
     243 -> 247         0.17431
     244 -> 246         0.47364
     244 -> 247        -0.24032

 Excited State   3:      Singlet-E      1.9229 eV  644.79 nm  f=0.0004  <S**2>=0.000
     237 -> 247         0.10082
     241 -> 247         0.11248
     243 -> 246        -0.17397
     243 -> 247        -0.36054
     244 -> 246         0.22006
     244 -> 247         0.46648

 Excited State   4:      Singlet-E      1.9229 eV  644.79 nm  f=0.0004  <S**2>=0.000
     238 -> 247         0.10082
     242 -> 247        -0.11248
     243 -> 246        -0.22006
     243 -> 247         0.46648
     244 -> 246        -0.17397
     244 -> 247         0.36054

 Excited State   5:      Singlet-B      2.0415 eV  607.31 nm  f=0.3592  <S**2>=0.000
     245 -> 246         0.69692

 Excited State   6:      Singlet-A      2.8370 eV  437.02 nm  f=0.0000  <S**2>=0.000
     245 -> 247         0.68324

 Excited State   7:      Singlet-E      3.3027 eV  375.40 nm  f=0.1307  <S**2>=0.000
     216 -> 246         0.11841
     241 -> 246         0.53109
     241 -> 247        -0.34589
     245 -> 248         0.12516

 Excited State   8:      Singlet-E      3.3027 eV  375.40 nm  f=0.1307  <S**2>=0.000
     217 -> 246         0.11841
     242 -> 246         0.53109
     242 -> 247         0.34589
     245 -> 249        -0.12516

 Excited State   9:      Singlet-E      3.5849 eV  345.85 nm  f=0.0023  <S**2>=0.000
     222 -> 247        -0.12923
     238 -> 246         0.38268
     238 -> 247         0.15033
     239 -> 246        -0.22310
     240 -> 246         0.21185
     245 -> 249        -0.37563

 Excited State  10:      Singlet-E      3.5849 eV  345.85 nm  f=0.0023  <S**2>=0.000
     223 -> 247        -0.12923
     237 -> 246         0.38268
     237 -> 247        -0.15032
     239 -> 246        -0.21185
```

| | |
|---|---|
| 240 -> 246 | -0.22309 |
| 245 -> 248 | -0.37563 |

***References for Supporting Information***

[i] Burla, M. C.; Caliandro, R.; Camalli, M.; Carrozzini, B.; Cascarano, G. L.; De Caro, L.; Giacovazzo, C.; Polidori, G.; Spagna, R. *J. Appl. Cryst.* **2005**, *38*, 381.

[ii] Sheldrick, G. M. *Acta Crystallogr. C, Struct. Chem.* **2015**, *71*, 3-8.

[iii] Yadokari-XG, Software for Crystal Structure Analyses, K. Wakita (2001); Release of Software (Yadokari-XG 2009) for Crystal Structure Analyses, Kabuto, C.; Akine, S.; Nemoto, T.; Kwon, E. *J. Cryst. Soc. Jpn.* **2009**, *51*, 218-224.

[iv] Krygowski, T. M. *J. Chem. Inf. Comput. Sci.* **1993**, *33*, 70-78.

[v] Furuyama, T.; Ogura, Y.; Yoza, K.; Kobayashi, N. *Angew. Chem. Int. Ed.* **2012**, *51*, 11110-11114.

[vi] Chauke, V.; Nyokong, T. *Inorg. Chim. Acta* **2010**, *363*, 3662-3669.

[vii] a) Becke, A. D. *Phys. Rev.* **1988**, *A38*, 3098-3100. b) Becke, A. D. *J. Chem. Phys.* **1993**, *98*, 1372-1377. c) Becke, A. D. *J. Chem. Phys.* **1993**, *98*, 5648-5652. d) Lee, C.; Yang, W.; Parr, R. G. *Phys. Rev.* **1988**, *B37*, 785-788.

[viii] *Gaussian 09*, Revision C.01, Frisch, M. J.; Trucks, G. W.; Schlegel, H. B.; Scuseria, G. E.; Robb, M. A.; Cheeseman, J. R.; Scalmani, G.; Barone, V.; Mennucci, B.; Petersson, G. A.; Nakatsuji, H.; Caricato, M.; Li, X.; Hratchian, H. P.; Izmaylov, A. F.; Bloino, J.; Zheng, G.; Sonnenberg, J. L.; Hada, M.; Ehara, M.; Toyota, K.; Fukuda, R.; Hasegawa, J.; Ishida, M.; Nakajima, T.; Honda, Y.; Kitao, O.; Nakai, H.; Vreven, T.; Montgomery, Jr., J. A.; Peralta, J. E.; Ogliaro, F.; Bearpark, M.; Heyd, J. J.; Brothers, E.; Kudin, K. N.; Staroverov, V. N.; Kobayashi, R.; Normand, J.; Raghavachari, K.; Rendell, A.; Burant, J. C.; Iyengar, S. S.; Tomasi, J.; Cossi, M.; Rega, N.; Millam, J. M.; Klene, M.; Knox, J. E.; Cross, J. B.; Bakken, V.; Adamo, C.; Jaramillo, J.; Gomperts,

R.; Stratmann, R. E.; Yazyev, O.; Austin, A. J.; Cammi, R.; Pomelli, C.; Ochterski, J. W.; Martin, R. L.; Morokuma, K.; Zakrzewski, V. G.; Voth, G. A.; Salvador, P.; Dannenberg, J. J.; Dapprich, S.; Daniels, A. D.; Farkas, Ö.; Foresman, J. B.; Ortiz, J. V.; Cioslowski, J.; Fox, D. J. Gaussian, Inc., Wallingford CT, 2009.

[ix] Andrae, D.; Haeussermann, U.; Dolg, M.; Stoll, H.; Preuss, H. *Thero. Chem. Acc.* **1990**, *77*, 123-141.

[x] a) Bauernschmitt, R. d.; Ahlrichs, R. *Chem. Phys. Lett.* **1996**, *256*, 454-464. b) Dreuw, A.; Head-Gordon, M. *Chem. Rev.* **2005**, *105*, 4009-4037.

[xi] Chai, J.-D.; Head-Gordon, M. *Phys. Chem. Chem. Phys.* **2008**, *10*, 6615-6620.

[xii] Tomasi, J.; Mennucci, B.; Cammi, R. *Chem. Rev.* **2005**, *105*, 2999-3094.
